# Leveraging laboratory biomarkers to predict urosepsis after upper urinary tract stone surgery: an explainable machine learning approach

**DOI:** 10.1186/s12911-025-03314-y

**Published:** 2025-12-20

**Authors:** Zuheng Wang, Xiao Li, Qin Li, Rongbin Zhou, Dongwei Pan, Zequn Su, Cunmeng Wei, Wenhao Lu, Fubo Wang

**Affiliations:** 1 Center for Genomic and Personalized Medicine, Guangxi key Laboratory for Genomic and Personalized Medicine, Guangxi Collaborative Innovation Center for Genomic and Personalized Medicine, University Engineering Research Center of Digital Medicine and Healthcare, Guangxi Medical University, Nanning, 530021 Guangxi China; 2 Department of Urology, the First Affiliated Hospital of Guangxi Medical University, Guangxi Medical University, Nanning, 530021 Guangxi China; 3 School of Life Sciences, Guangxi Medical University, Nanning, 530021 Guangxi China; 4 Department of Hematology, The First Affiliated Hospital of Guangxi Medical University, Guangxi Medical University, Nanning, 530021 Guangxi China; 5 Department of Urology, The Second Affiliated Hospital of Fujian Medical University, Quanzhou, 362000 China; 6 Department of Urology, Guangxi Hospital Division of the First Affiliated Hospital, Sun Yat-Sen University, Nanning, 530022 Guangxi China; 7 School of Public Health, Guangxi Medical University, Nanning, 530021 Guangxi China

**Keywords:** Urosepsis, Calculi, Percutaneous nephrolithotomy, Retrograde intrarenal surgery, Machine learning, Artificial intelligence

## Abstract

**Objective:**

Urosepsis is a leading cause of perioperative mortality after upper urinary tract stone surgery. This study aimed to develop a simple and accurate predictive model for postoperative sepsis by integrating laboratory parameters using machine learning (ML) and the Shapley Additive Explanations (SHAP) algorithm.

**Methods:**

Data from 7,464 patients were analyzed, including 155 pre- and postoperative features. Key variables were selected using Least Absolute Shrinkage and Selection Operator (LASSO) regression and correlation analysis. Eight ML algorithms were employed to develop the predictive model using 10-fold cross-validation. The model’s performance was assessed using Receiver Operating Characteristic curve, learning curve, calibration plot, and decision curve. The SHAP algorithm was employed to analyze variable importance. Finally, the model was converted into a publicly accessible application.

**Results:**

116 out of 155 variables (74.84%) differed significantly between the urosepsis (*n* = 622, 8.33%) and non-urosepsis groups (*n* = 6,842, 91.67%). LASSO regression identified eight predictive variables: postoperative IL-6, SAA, PCT/ALB, NLPR, PT, ALB, HCT, and neutrophil. Light Gradient Boosting Machine achieved the best performance, with AUCs of 1.0, 0.90, and 0.88 for the training, validation, and test cohorts, respectively. A good model fit, strong calibration, and positive clinical utility were confirmed by the learning curve, calibration plot, and decision curve. Postoperative PCT/ALB, neutrophil, IL-6, ALB, and PT were identified as key predictors by the SHAP algorithm. A publicly accessible web application was developed to facilitate both patients and physicians (https://www.xsmartanalysis.com/model/list/predict/model/html?mid=29648&symbol=71lphm763138mj895nd8).

**Conclusions:**

A robust, interpretable model based on postoperative laboratory biomarkers was successfully developed and validated. This model exhibits excellent predictive performance and clinical utility, and the integration of SHAP analysis provides transparent insights into the key drivers of urosepsis, offering a practical tool for early risk stratification and personalized intervention in clinical practice.

**Trial registration:**

Prospective registered in the Chinese Clinical Trial Registry (trial registration number: ChiCTR2400079409, date of registration: 2024-01-03).

**Clinical trial registration:**

This study was registered with the Chinese Clinical Trial Registry on January 3, 2024 (ChiCTR2400079409, http://www.chictr.org.cn/).

**Supplementary Information:**

The online version contains supplementary material available at 10.1186/s12911-025-03314-y.

## Introduction

Urolithiasis is a common condition in urology, with a lifetime prevalence of 10–20% and an annual incidence of approximately 1% [1], particularly with upper urinary tract stones being more common [2]. With advances of minimally invasive techniques, procedures such as percutaneous nephrolithotomy (PCNL), retrograde intrarenal surgery (RIRS), and ureteroscopic lithotripsy (URL) have become the first-line treatments for upper urinary tract stones [3]. Minimally invasive techniques offer advantages such as minimal trauma, high stone clearance rates, and rapid postoperative recovery. However, they may lead to complications such as bleeding and infection [4]. Among these, urosepsis is the most severe complication and a common cause of perioperative mortality [5]. Urosepsis results from the invasion of microorganisms and their toxins into the bloodstream, leading to systemic inflammatory response syndrome and organ dysfunction, even death in severe cases [6]. Urosepsis can escalate to septic shock, with a mortality rate as high as 66%–80% [7].

Early identification and effective control of urosepsis are crucial for improving patient outcomes and survival rates. According to international guidelines for the management of sepsis and septic shock, antibiotics should be administered within the first hour of recognition [8]. Studies indicate that each one-hour delay in antibiotic administration increases the risk of septic shock by 1.8%, and overall mortality rises by 3–13% in patients with sepsis [9]. Blood culture is essential for the diagnosis of sepsis, especially in the neonatal field, where blood culture (including peripheral and umbilical cord blood) is considered the “gold standard” [10]. However, blood culture has notable limitations, including a long turnaround time (48–72 hours) and low sensitivity [11]. Emerging biomarkers offer potential for earlier identification of sepsis and may assist in identifying causative pathogens. To date, hundreds of sepsis-related biomarkers have been studied, including procalcitonin (PCT), C-reactive protein (CRP), lactate, and various cytokines, among which PCT and CRP are the most widely used in clinical practice [12]. In healthy individuals, PCT is secreted by neuroendocrine cells of the thyroid, but during infection, it can be released by a variety of cell types [13]. PCT levels rise within 2–4 hours after infection and have better diagnostic performance than other inflammatory markers [14]. Nonetheless, Dragoescu et al. found that the area under the receiver operating characteristic curve (AUC) of PCT for diagnosing urosepsis was only 0.743 [15]. The AUC of CRP for sepsis diagnosis is 0.77, which is comparable to the diagnostic performance of PCT [16]. Cytokines are significantly elevated in sepsis patients and participate in the regulation of inflammatory responses, but their diagnostic efficacy is inferior to that of PCT and CRP [17]. These limitations highlight the urgent need for more accurate and timely methods for the early diagnosis of urosepsis.

To address these challenges, we developed a Machine learning (ML) model to predict urosepsis based on large-scale clinical data from patients with upper urinary tract stones. To enhance model interpretability and support clinical decision-making, we applied the SHapley Additive exPlanations (SHAP) algorithm to explain the model’s predictions.

## Materials and methods

### Source of data

This real-world, retrospective study was conducted at the Sixth Affiliated Hospital of Guangxi Medical University, China (The First People’s Hospital of Yulin). Clinical data were collected from the electronic medical records of 7,464 patients between January 2018 and June 2023.

The study protocol was approved by the Medical Ethics Committee of Guangxi Medical University (KY20230266) and registered with the Chinese Clinical Trial Registry on January 3, 2024 (ChiCTR2400079409). Given the retrospective design and anonymization of patient data, the requirement for informed consent was waived by the Ethics Committee. This study adheres to the STROBE statement (Supplementary Table 1) and complied with the principles of the Declaration of Helsinki.

### Study patients

Inclusion criteria were as follows: (1) age ≥ 18 years; (2) diagnosis of upper urinary tract stones confirmed by ultrasound or computed tomography, followed by minimally invasive surgery (PCNL, RIRS or URL); (3) availability of complete clinical records; (4) diagnosis of urosepsis (experimental group) or absence of urosepsis (control group). Exclusion criteria were: (1) simultaneous performance of more than one surgical procedure; (2) presence of infectious lesions unrelated to urolithiasis. All urosepsis cases were diagnosed within 48 hours after surgery.

### Study predictors

Multimodal clinical parameters of eligible participants before and after operation were extracted using extract-transform-load (ETL) tools. Clinical variables recorded for risk factor analysis and model development included age, gender, height, weight, history of hypertension and diabetes, and imaging reports (ultrasound and non-contrast computed tomography). Barthel Index scores, venous thromboembolism (VTE) risk assessments, Morse Fall Scale scores, information on whether patients had diabetes or hypertension, and the American Society of Anesthesiologists (ASA) physical status classification were also collected. In addition, laboratory test data obtained immediately before and after surgery were extracted.

In addition, we extracted laboratory test data obtained within 24 hours before surgery (preoperative laboratory data) and within 6 hours after surgery (postoperative laboratory data), distinguished by adding the prefixes “p-”. Given the diagnostic value of systemic immune-inflammatory biomarkers in sepsis, we analyzed haematological biomarkers (e.g., white blood cell count, neutrophil count, hematocrit), biochemical biomarkers (e.g., albumin, β2-microglobulin, cystatin C), inflammatory biomarkers (e.g., PCT, CRP, IL-6, SAA), and immune-inflammatory ratios (e.g., neutrophil-to-lymphocyte ratio, platelet-to-lymphocyte ratio).

In total, 155 clinical multimodal features were included in this study.

### Feature selection

Initially, 20% of patients were randomly sampled from the entire cohort to serve as the test cohort, while the remaining 80% were randomly split into the training cohort and validation cohort at a ratio of 8:2. Feature selection was exclusively performed within the training cohort.

The test set was strictly held out and not involved in any step of feature selection or model training. We analyzed the distribution of 155 clinical multimodal features between the experimental (urosepsis) and control (non-urosepsis) groups in the training cohort. For normally distributed continuous variables, Student’s t-test was employed to compare intergroup differences. For non-normally distributed continuous variables, the Mann-Whitney U test was utilized. Categorical variables were compared via the chi-square test. Only variables with statistically significant differences (*p* < 0.05) were retained for subsequent analysis.

Next, we applied Least Absolute Shrinkage and Selection Operator (LASSO) regression with 10-fold cross-validation to identify parameters with non-zero coefficients. To assess multicollinearity among the selected features, Pearson correlation analysis was conducted, and features with an absolute correlation coefficient greater than 0.7 with any other feature were excluded.

### Machine learning process

Following feature selection, eight ML models were developed: Gradient Boosting Decision Tree (GBDT), Light Gradient Boosting Machine (LightGBM), Adaptive Boosting (AdaBoost), Random Forest (RF), eXtreme Gradient Boosting (XGBoost), Gaussian Naive Bayes (GNB), Logistic Regression and Support Vector Machine (SVM). Each model was trained on the training dataset and subsequently evaluated on the validation cohort.

GBDT excels at capturing non-linear relationships and biomarker interactions critical for complex clinical outcome prediction, while LightGBM offers high efficiency and scalability to handle large-scale datasets without compromising performance [18]. XGBoost enhances model stability in real-world settings through built-in regularization and missing value handling, addressing common challenges with clinical data completeness [19]. RF serves as a reliable baseline, being inherently resistant to overfitting and outliers, and AdaBoost prioritizes misclassified samples to improve detection of rare urosepsis cases [20]. GNB provides a simple, interpretable baseline to assess the added value of more complex algorithms, while Logistic Regression enables straightforward clinical interpretation via direct probability outputs and regression coefficients [21]. Finally, SVM complements tree-based algorithms by leveraging kernel methods to capture subtle patterns between biomarkers, further enriching the model comparison landscape [22].

### Model performance evaluation

The AUC and Area Under the Precision-Recall Curve (PR-AUC) were the primary metric used to assess model performance, and DeLong’s test was applied to compare the AUCs of different algorithms. In addition, we calculated accuracy, sensitivity, specificity, positive predictive value (PPV), and negative predictive value (NPV) to comprehensively evaluate model performance. After determining the optimal features and hyperparameters, the final model was retrained using the combined training and validation cohort, and its performance was ultimately evaluated on the independent test set.

Learning curves were generated to illustrate changes in model performance with increasing sample size, allowing for the detection of potential underfitting or overfitting. Calibration plots, based on the Hosmer-Lemeshow test, were used to assess the agreement between predicted probabilities and observed outcomes. To address the interpretability challenge commonly associated with ML models, the SHAP algorithm was employed to quantify and visualize the contribution of each feature to model predictions, thereby enhancing transparency and aiding clinical interpretation.

### Statistical analysis

All data analyses were performed using Python software (version 3.8) and R (version 4.2.3). Continuous variables were summarized as mean ± standard deviation for normally distributed data, or as median and interquartile range for non-normally distributed data. Student’s t-test was used for normally distributed continuous variables, while the Mann-Whitney U test was applied for non-normally distributed ones. Diagnostic metrics, including sensitivity, specificity, PPV, and NPV, were derived from the confusion matrix. A two-tailed *P*-value of less than 0.05 was considered statistically significant.

## Results

### Clinical characteristics of patients

The study flowchart is shown in Supplementary Figure 1. A total of 7,464 patients with upper urinary tract stones who underwent surgery were included in this study. Patients were divided into the experimental group (urosepsis, *n* = 622) and the control group (non-urosepsis, *n* = 6,842). Among the 622 patients who developed urosepsis, 527 (84.7%) stabilized after receiving appropriate treatment, including fluid resuscitation, anti-infective therapy, and administration of norepinephrine. 95 patients (15.3%) were transferred to the Intensive Care Unit for further treatment, and 3 patients (0.5%) died. The remaining patients showed improvement and were discharged.

Of the total study population, 4,486 (60.1%) were male and 2,978 (39.9%) were female. Notably, females were more prevalent in the urosepsis group (*p* < 0.001, Table 1). The urosepsis group also had a significantly higher proportion of patients undergoing percutaneous nephrolithotomy (57.88% vs. 38.53%, *p* < 0.001) and more frequent endoscopic procedures (*p* < 0.001). No significant differences were observed in the prevalence of diabetes or hypertension between the two groups (*p* > 0.05). Out of the 155 clinical features analyzed, 116 parameters (74.84%) showed significant differences between the groups (*p* < 0.05). We also collected several clinical scores, including the Barthel Index, VTE, Morse, and ASA scores. In the urosepsis group, VTE and ASA scores were higher, while the Barthel Index was lower (*p* < 0.05), with no significant difference in the Morse score (*p* > 0.05). All patients were randomly divided into a training cohort (*n* = 4776, 64%), validation cohort (*n* = 1,195, 16%) and test cohort (*n* = 1,493, 20%).

**Table 1:** Clinical characteristics of patients

Variables	ALL (n = 7464)	Non-urosepsis (n = 6842)	Urosepsis (n = 622)	*P***-value**
BMI^†^	23.74 (21.48, 25.95)	23.80 (21.48, 25.97)	23.26 (20.83, 25.52)	< 0.001
Sex^§^				< 0.001
Female	2978 (39.90%)	2622 (38.32%)	356 (57.23%)	
Male	4486 (60.10%)	4220 (61.68%)	266 (42.77%)	
Type of operation^§^				< 0.001
PCNL	2996 (40.14%)	2636 (38.53%)	360 (57.88%)	
RIRS	1910 (25.59%)	1786 (26.10%)	124 (19.94%)	
URL	2558 (34.27%)	2420 (35.37%)	138 (22.19%)	
ASA^§^				0.004
1	1103 (14.89%)	1037 (15.28%)	66 (10.65%)	
2	6144 (82.96%)	5605 (82.60%)	539 (86.94%)	
3	156 (2.11%)	142 (2.09%)	14 (2.26%)	
4	3 (0.04%)	2 (0.03%)	1 (0.16%)	
Barthel index^†^	40.00 (30.00, 85.00)	40.00 (30.00, 85.00)	35.00 (25.00, 80.00)	0.015
VTE^†^	2.00 (1.00, 3.00)	2.00 (1.00, 3.00)	2.00 (1.00, 3.00)	0.007
U_LEU^†^	1.00 (0.00, 3.00)	1.00 (0.00, 3.00)	2.00 (1.00, 4.00)	< 0.001
U_NIT^§^				< 0.001
0	7106 (95.20%)	6557 (95.83%)	549 (88.26%)	
+	160 (2.14%)	126 (1.84%)	34 (5.47%)	
++	198 (2.65%)	159 (2.32%)	39 (6.27%)	
U_PRO†	0.00 (0.00, 1.00)	0.00 (0.00, 1.00)	0.00 (0.00, 1.00)	0.001
Times of operation†	1.00 (1.00, 2.00)	1.00 (1.00, 2.00)	1.00 (1.00, 2.00)	< 0.001
SG†	1.01 (1.00, 1.01)	1.01 (1.00, 1.01)	1.01 (1.00, 1.01)	< 0.001
PH †	6.00 (6.00, 6.50)	6.00 (6.00, 6.50)	6.50 (6.00, 7.00)	0.003
SED_WBC†	45.55 (14.00, 168.02)	42.00 (13.00, 151.00)	124.50 (29.05, 496.50)	< 0.001
SED_bacteria†	24.90 (9.00, 90.00)	23.00 (9.00, 84.00)	43.50 (15.00, 219.00)	< 0.001
U_conductivity†	12.30 (8.70, 15.90)	12.40 (8.80, 16.00)	10.80 (7.90, 14.17)	< 0.001
WBC†	6.96 (5.84, 8.36)	6.92 (5.83, 8.30)	7.30 (6.08, 8.83)	< 0.001
Neut%†	60.30 (54.40, 66.40)	60.20 (54.30, 66.20)	61.45 (54.82, 68.20)	0.002
Lymph%†	28.00 (22.40, 33.30)	28.10 (22.60, 33.30)	26.90 (20.90, 32.80)	0.003
Baso%†	0.60 (0.40, 0.80)	0.60 (0.40, 0.80)	0.60 (0.40, 0.80)	0.02
Neut†	4.14 (3.29, 5.25)	4.12 (3.28, 5.20)	4.44 (3.38, 5.71)	< 0.001
Mono†	0.48 (0.38, 0.60)	0.48 (0.38, 0.60)	0.49 (0.38, 0.64)	0.014
RBC†	4.56 (4.15, 5.02)	4.58 (4.16, 5.03)	4.38 (3.97, 4.81)	< 0.001
HGB†	129.00 (117.00, 142.00)	130.00 (118.00, 143.00)	122.00 (109.00, 135.00)	< 0.001
HCT†	0.39 (0.36, 0.43)	0.40 (0.36, 0.43)	0.37 (0.34, 0.41)	< 0.001
MCV†	87.60 (83.40, 90.90)	87.70 (83.50, 91.00)	86.80 (82.65, 90.30)	0.003
MCH†	29.20 (27.30, 30.50)	29.21 (27.40, 30.50)	28.70 (26.60, 30.16)	< 0.001
MCHC†	331.00 (320.00, 339.00)	331.00 (321.00, 339.00)	329.00 (318.00, 336.15)	< 0.001
RDW†	12.70 (12.10, 13.64)	12.70 (12.10, 13.60)	13.00 (12.30, 14.10)	< 0.001
PLT†	266.00 (225.00, 315.00)	264.00 (224.00, 312.00)	287.00 (241.00, 342.00)	< 0.001
MPV†	9.70 (9.20, 10.20)	9.70 (9.20, 10.20)	9.60 (9.10, 10.10)	0.001
Plateletcrit†	0.26 (0.22, 0.30)	0.26 (0.22, 0.30)	0.28 (0.24, 0.32)	< 0.001
eGFR†	73.94 (55.50, 89.35)	74.04 (55.83, 89.49)	72.09 (50.79, 88.73)	0.026
† HCO3	23.80 (22.10, 25.40)	23.80 (22.20, 25.40)	23.50 (21.42, 25.00)	< 0.001
β2-Mg†	2.27 (1.87, 2.95)	2.26 (1.86, 2.90)	2.51 (2.03, 3.62)	< 0.001
CysC†	1.13 (0.99, 1.38)	1.12 (0.98, 1.37)	1.19 (1.01, 1.53)	< 0.001
TP†	70.50 (67.30, 73.90)	70.40 (67.20, 73.70)	71.45 (68.10, 75.30)	< 0.001
ALB†	40.00 (37.80, 42.10)	40.10 (37.90, 42.20)	39.40 (36.70, 41.40)	< 0.001
GLB†	30.30 (27.60, 33.50)	30.10 (27.50, 33.30)	32.40 (29.00, 36.10)	< 0.001
ALB/GLB†	1.30 (1.20, 1.50)	1.30 (1.20, 1.50)	1.20 (1.10, 1.40)	< 0.001
BIL†	7.50 (5.40, 10.30)	7.60 (5.50, 10.40)	6.80 (4.90, 9.40)	< 0.001
DBIL†	3.50 (2.60, 4.60)	3.50 (2.70, 4.60)	3.20 (2.40, 4.30)	< 0.001
IBil†	4.00 (2.70, 5.72)	4.00 (2.76, 5.80)	3.60 (2.30, 5.18)	< 0.001
ALP†	72.00 (61.00, 86.00)	71.00 (60.00, 86.00)	76.00 (63.00, 90.00)	< 0.001
CRP†	2.51 (1.12, 7.06)	2.43 (1.09, 6.70)	3.81 (1.62, 11.57)	< 0.001
PT†	10.90 (10.40, 11.50)	10.90 (10.40, 11.50)	11.00 (10.50, 11.50)	0.007
INR†	0.95 (0.90, 1.00)	0.95 (0.90, 1.00)	0.95 (0.91, 1.00)	0.013
PTA†	114.00 (101.00, 127.00)	114.00 (101.00, 128.00)	112.00 (98.00, 125.00)	< 0.001
TT†	18.62 (17.80, 19.50)	18.62 (17.80, 19.50)	18.50 (17.60, 19.40)	0.001
PF†	3.10 (2.62, 3.88)	3.08 (2.60, 3.83)	3.43 (2.87, 4.61)	< 0.001
NLR†	2.16 (1.64, 2.94)	2.14 (1.64, 2.91)	2.29 (1.66, 3.28)	0.002
PLR†	141.27 (111.16, 182.38)	140.52 (110.91, 180.95)	151.15 (117.12, 198.67)	< 0.001
dNLR†	1.52 (1.19, 1.98)	1.52 (1.19, 1.96)	1.60 (1.22, 2.15)	0.002
SII†	576.15 (408.94, 837.10)	570.11 (406.72, 819.28)	652.61 (447.08, 1024.52)	< 0.001
AISI†	273.60 (174.02, 451.07)	269.27 (172.40, 441.30)	314.19 (197.34, 571.32)	< 0.001
SIRI†	1.01 (0.69, 1.58)	1.00 (0.69, 1.56)	1.10 (0.72, 1.85)	0.001
LCR†	0.76 (0.26, 1.72)	0.79 (0.27, 1.77)	0.51 (0.15, 1.22)	< 0.001
CRP/ALB†	0.06 (0.03, 0.18)	0.06 (0.03, 0.17)	0.10 (0.04, 0.30)	< 0.001
WBC-p_WBC†	0.86 (0.68, 1.08)	0.87 (0.70, 1.08)	0.67 (0.50, 1.00)	< 0.001
p_CRP†	2.47 (0.87, 7.89)	2.24 (0.81, 6.75)	10.35 (2.45, 41.30)	< 0.001
p_SAA†	10.00 (7.00, 25.25)	10.00 (6.00, 21.00)	32.00 (10.00, 285.00)	< 0.001
p_PCT†	0.05 (0.03, 0.09)	0.05 (0.03, 0.08)	0.21 (0.07, 4.99)	< 0.001
p_IL_6†	14.62 (6.53, 37.03)	12.88 (6.17, 29.91)	79.22 (20.50, 505.30)	< 0.001
p_WBC†	8.14 (6.43, 10.33)	8.01 (6.40, 9.97)	12.14 (6.96, 14.70)	< 0.001
p_Neut%†	71.60 (62.30, 81.70)	70.90 (61.90, 80.40)	81.70 (69.30, 89.30)	< 0.001
p_Lymph%†	19.70 (12.50, 27.60)	20.30 (13.20, 28.20)	11.90 (6.35, 20.85)	< 0.001
p_Mono%†	5.60 (3.50, 7.10)	5.60 (3.70, 7.10)	4.80 (2.50, 7.00)	< 0.001
p_Eos%†	1.40 (0.60, 2.70)	1.50 (0.60, 2.80)	0.80 (0.20, 2.20)	< 0.001
p_Baso%†	0.40 (0.30, 0.60)	0.40 (0.30, 0.60)	0.30 (0.20, 0.40)	< 0.001
p_Neut†	5.67 (4.16, 7.90)	5.56 (4.14, 7.56)	9.44 (5.01, 12.73)	< 0.001
p_Lymph†	1.51 (1.03, 2.10)	1.54 (1.06, 2.12)	1.15 (0.72, 1.73)	< 0.001
p_Mono†	0.43 (0.27, 0.59)	0.43 (0.27, 0.58)	0.49 (0.23, 0.78)	< 0.001
p_Eos†	0.11 (0.05, 0.21)	0.11 (0.05, 0.21)	0.08 (0.03, 0.19)	< 0.001
p_Baso†	0.03 (0.02, 0.04)	0.03 (0.02, 0.04)	0.03 (0.02, 0.05)	0.048
p_NRBC†	0.00 (0.00, 0.00)	0.00 (0.00, 0.00)	0.00 (0.00, 0.00)	< 0.001
p_NRBC/RBC†	0.00 (0.00, 0.00)	0.00 (0.00, 0.00)	0.00 (0.00, 0.00)	< 0.001
p_RBC†	4.35 (3.89, 4.82)	4.38 (3.94, 4.85)	3.95 (3.43, 4.43)	< 0.001
p_HGB†	123.00 (110.00, 137.00)	124.00 (111.00, 137.00)	110.00 (95.00, 125.00)	< 0.001
p_HCT†	0.37 (0.34, 0.41)	0.38 (0.34, 0.41)	0.33 (0.29, 0.38)	< 0.001
p_MCV†	87.60 (83.20, 91.00)	87.70 (83.40, 91.20)	86.50 (81.50, 90.15)	< 0.001
p_MCH†	29.30 (27.40, 30.60)	29.30 (27.50, 30.60)	28.80 (26.75, 30.10)	< 0.001
p_RDW†	12.70 (12.00, 13.70)	12.60 (12.00, 13.60)	13.10 (12.40, 14.30)	< 0.001
p_BUA†	263.00 (198.00, 337.98)	264.05 (198.60, 338.80)	253.80 (186.52, 328.83)	0.009
p_BUN†	4.20 (3.30, 5.40)	4.19 (3.30, 5.30)	4.30 (3.20, 5.90)	0.038
p_Scr†	94.00 (76.00, 119.00)	93.00 (76.00, 118.00)	99.00 (76.00, 138.50)	< 0.001
p_eGFR†	75.41 (55.90, 94.69)	76.10 (56.79, 94.93)	65.36 (41.17, 89.47)	< 0.001
p_HCO3†	23.10 (21.20, 24.90)	23.20 (21.30, 25.00)	21.90 (20.10, 23.80)	< 0.001
p_β2-MG†	2.12 (1.68, 2.85)	2.08 (1.65, 2.77)	2.71 (2.03, 4.13)	< 0.001
p_TP†	65.50 (61.50, 69.30)	65.60 (61.70, 69.30)	62.95 (58.32, 68.90)	< 0.001
p_ALB†	36.30 (33.80, 38.70)	36.50 (34.10, 38.80)	33.60 (30.50, 36.40)	< 0.001
p_GLB†	28.80 (26.10, 31.90)	28.80 (26.20, 31.80)	29.60 (25.90, 33.20)	0.004
p_ALB/GLB†	1.30 (1.10, 1.40)	1.30 (1.10, 1.40)	1.10 (1.00, 1.30)	< 0.001
p_DBIL†	4.60 (3.50, 6.10)	4.60 (3.50, 6.00)	5.00 (3.80, 6.80)	< 0.001
p_IBil†	5.40 (3.60, 7.80)	5.50 (3.80, 7.90)	4.50 (2.90, 6.68)	< 0.001
p_GPT†	14.00 (10.00, 21.00)	14.00 (10.00, 20.70)	15.00 (10.00, 22.00)	0.013
p_GOT†	16.20 (14.00, 20.30)	16.00 (13.90, 20.00)	19.00 (15.00, 27.30)	< 0.001
p_GGT†	25.00 (17.00, 40.00)	24.00 (16.00, 39.00)	33.20 (20.00, 56.53)	< 0.001
p_TBA†	1.80 (1.00, 3.30)	1.70 (1.00, 3.25)	2.30 (1.20, 4.40)	< 0.001
p_K†	3.83 (3.57, 4.11)	3.85 (3.59, 4.13)	3.68 (3.42, 3.98)	< 0.001
p_Ca†	2.18 (2.09, 2.26)	2.18 (2.10, 2.26)	2.14 (2.04, 2.23)	< 0.001
p_PT†	11.70 (11.20, 12.30)	11.70 (11.20, 12.30)	12.20 (11.67, 13.12)	< 0.001
p_INR†	1.02 (0.97, 1.07)	1.02 (0.97, 1.07)	1.06 (1.02, 1.15)	< 0.001
p_PTA†	101.00 (91.00, 114.00)	103.00 (93.00, 115.00)	94.00 (78.50, 105.00)	< 0.001
p_TT†	18.50 (17.60, 19.50)	18.50 (17.70, 19.50)	18.10 (17.10, 19.42)	0.001
p_APTT†	26.50 (23.50, 29.60)	26.40 (23.50, 29.30)	27.90 (23.48, 32.82)	< 0.001
p_PF†	3.12 (2.53, 3.91)	3.09 (2.53, 3.85)	3.41 (2.64, 4.62)	0.001
p_NLR†	3.63 (2.27, 6.50)	3.50 (2.21, 6.09)	6.70 (3.33, 14.23)	< 0.001
p_PLR†	166.01 (117.09, 247.82)	162.44 (115.78, 240.91)	216.24 (143.54, 333.03)	< 0.001
p_LMR†	3.98 (2.61, 6.06)	4.09 (2.73, 6.13)	2.66 (1.44, 4.73)	< 0.001
p_dNLR†	2.52 (1.65, 4.44)	2.44 (1.63, 4.11)	4.46 (2.26, 8.32)	< 0.001
p_NLPR†	0.01 (0.01, 0.03)	0.01 (0.01, 0.03)	0.03 (0.01, 0.06)	< 0.001
p_SII†	924.30 (550.82, 1685.43)	890.14 (539.67, 1564.70)	1768.98 (863.31, 3303.84)	< 0.001
p_AISI†	337.79 (188.23, 654.79)	322.61 (185.01, 598.17)	804.62 (296.30, 1811.45)	< 0.001
p_LCR†	0.59 (0.16, 1.78)	0.66 (0.19, 1.94)	0.10 (0.02, 0.49)	< 0.001
p_SIRI†	1.32 (0.77, 2.47)	1.27 (0.76, 2.28)	3.08 (1.14, 7.31)	< 0.001
p_CRP/ALB†	0.07 (0.02, 0.23)	0.06 (0.02, 0.19)	0.32 (0.08, 1.30)	< 0.001
p_PCT/ALB†	< 0.01 ( < 0.01, < 0.01)	< 0.01 ( < 0.01, < 0.01)	0.01 ( < 0.01, 0.11)	< 0.001

†Data are shown as Median (Q1–Q3) and analyzed using the Mann-Whitney U test

§Data are shown as N (%) and analyzed using the Chi-square test

U_LEU, urine leukocytes; U_NIT, urine nitrite; U_PRO, urine protein; SED_WBC, sediment white blood cells; SED_bacteria, sedimen, bacteria; WBC, white blood count; Neut%, neutrophil percentage; Neut, absolute neutrophil count; Mono, monocyte count; RDW, red, cell distribution width; β2 MG, β2 microglobulin; CysC, cystatin C; TP, total protein; GLB, globulin; ALP, alkaline phosphatase; GGT, gamma-glutamyl transferase; SAA, Serum amyloid A; PCT, Procalcitonin; IL-6, Interleukin-6; CRP, C-reactive protein; PT, prothrombin, time; INR, international normalized ratio; PF, partial thromboplastin time; SED_EC, sediment erythrocytes; Lymph%, lymphocyte, percentage; HGB, hemoglobin; HCT, hematocrit; MCH, mean corpuscular hemoglobin; eGFR, estimated glomerular filtration rate; ALB, albumin; IBil, indirect bilirubin; NLR, neutrophil-to-lymphocyte ratio; dNLR, derived NLR; PLR, platelet-to-lymphocyte ratio; LMR, lymphocyte-to-monocyte ratio; ELR, eosinophil-to-lymphocyte ratio; NLPR, neutrophil-lymphocyte-platelet ratio; SII, systemic, immune-inflammation index; AISI, aggregate index of systemic inflammation; SIRI, systemic inflammation response index; LCR, lymphocyte-to-CRP ratio; CRP/ALB, CRP-to-albumin ratio; p_, postoperative.

### Feature selection

**Fig. 1:**
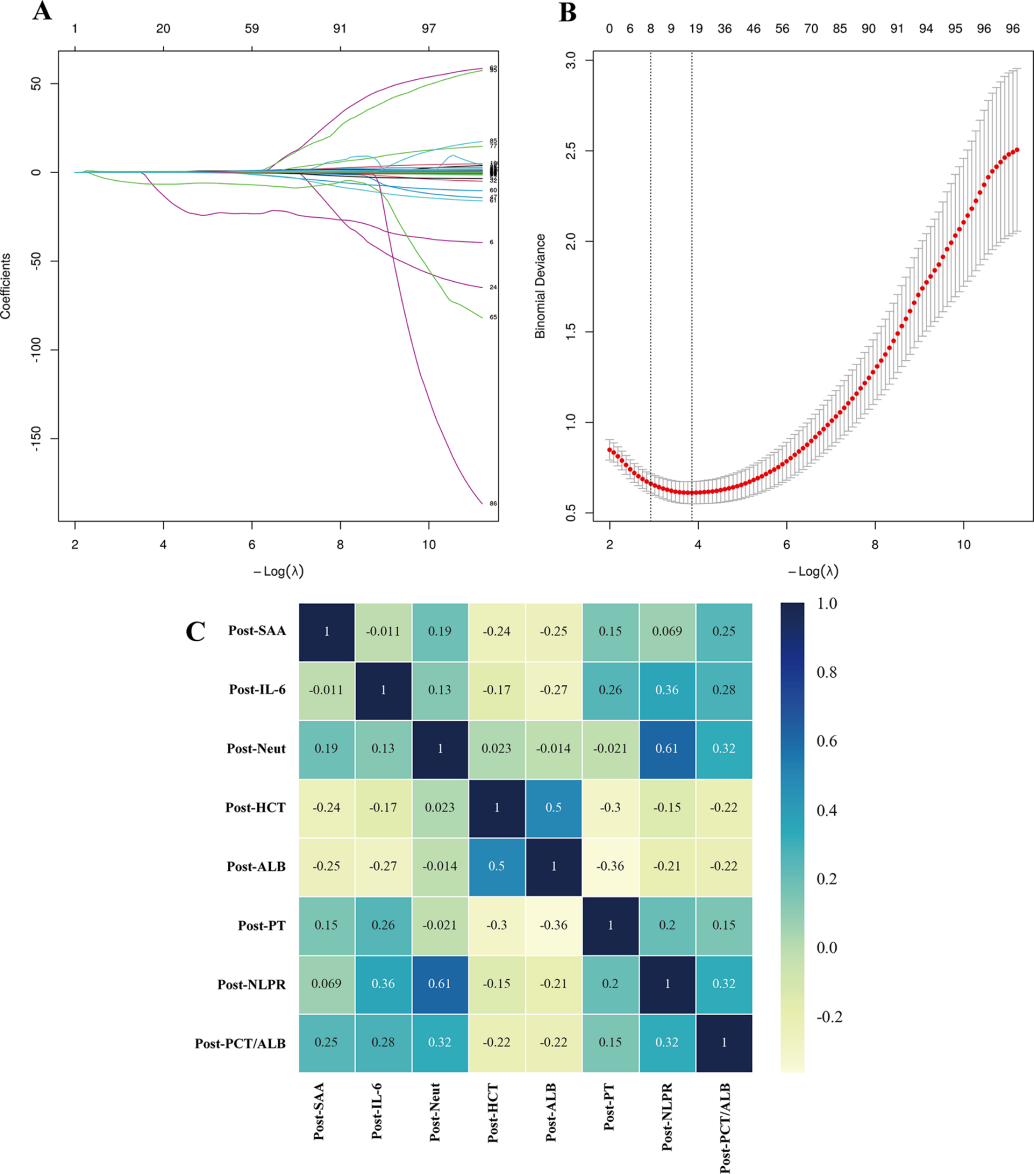
Lasso regression and Pearson correlation analysis. **A**. Coefficient path plot of lasso regression analysis. **B**. Cross-validation curve of lasso regression analysis. **C**. Pearson correlation heatmap. Post-SAA, postoperative serum amyloid a; post-PCT, postoperative procalcitonin; post-IL-6, postoperative interleukin-6; post-WBC, postoperative white blood cell; post-neut, postoperative Neutrophil; post-HCT, postoperative Hematocrit; post-β2-MG, postoperative β2-Microglobulin; post- ALB, postoperative Albumin; post-CRP/ALB, postoperative C-reactive protein/albumin

Feature selection was exclusively performed within the training cohort. A total of 106 parameters in the training cohort showed statistical differences (Supplementary Table 2). Given the large number of diverse parameters, we aimed to eliminate redundant features and enhance the stability of our predictive model. To this end, we employed LASSO regression to identify the most predictive variables from the high-dimensional clinical dataset. LASSO regression is a widely utilized technique for analyzing high-dimensional data, as it mitigates overfitting while preserving the most informative features. Using 10-fold cross-validation, we determined the optimal λ value to be 0.045, corresponding to the minimum standard error. This process resulted in the selection of eight non-zero variables: interleukin-6 (IL-6), serum amyloid A (SAA), albumin (ALB), PCT/ALB, neutrophil-lymphocyte-platelet ratio (NLPR), Prothrombin Time (PT), hematocrit (HCT), and neutrophils (Neut) (Fig. 1A–B, Supplementary Table 3).

To further assess potential multicollinearity, we conducted a Pearson correlation analysis. The Pearson correlation coefficient (R) was used to quantify the strength of linear relationships between continuous variables. High correlations (|R| > 0.7) may indicate multicollinearity, potentially leading to unstable regression coefficient estimates. Our analysis indicated that all selected variables exhibited only weak to moderate correlations (|R| < 0.7), as shown in Fig. 1Cand Supplementary Tables 4–5.

### Performance comparison of ML algorithms

Using the previously selected variables, we constructed a urosepsis prediction model. Baseline characteristics for both cohorts are presented in Supplementary Table 6. The model was trained on the training cohort, hyperparameters were tuned using the validation cohort, and final performance was assessed on the test cohort. Eight ML algorithms were evaluated: GBDT, LightGBM, AdaBoost, RF, XGBoost, GNB, Logistic Regression and SVM. The predictive performance of each model was assessed using AUC.

**Fig. 2:**
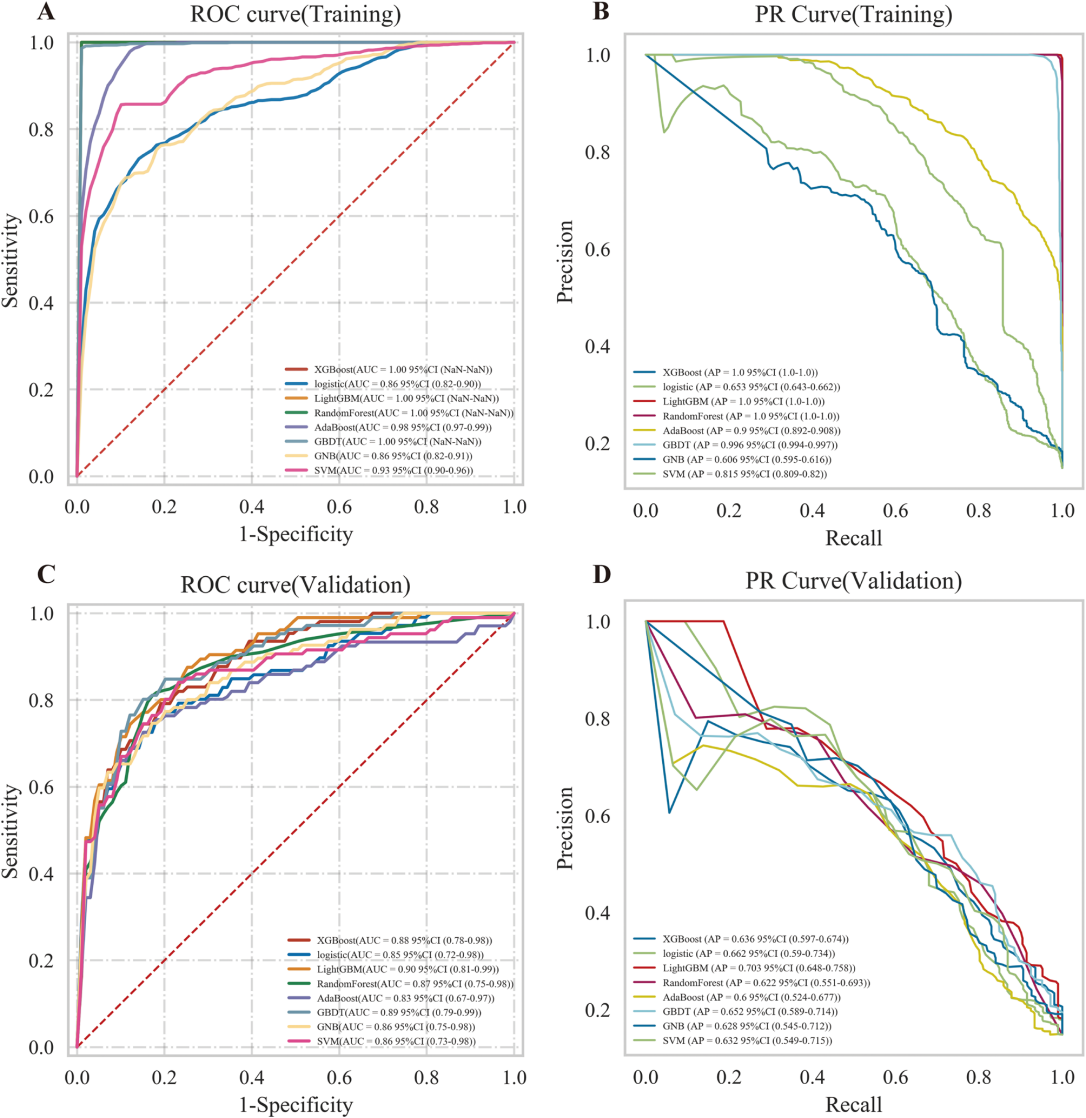
ROC curves of ml algorithms. **A**. ROC curves of the training cohort. **B**. ROC curves of the validation cohort. ROC, receiver operating characteristics; AUC, area under ROC; GBDT, gradient boosting decision Tree; LightGBM, light gradient boosting Machine; AdaBoost, adaptive Boosting; XGBoost, eXtreme gradient Boosting; GNB, Gaussian Naive Bayes; SVM, support vector machine

Following 10-fold cross-validation, LightGBM, RF, GBDT and XGBoost demonstrated the highest performance in the training cohort, each achieving an AUC of 1.00. Furthermore, their accuracy, sensitivity, specificity, PPV, and NPV and PR-AUC all nearly reached 1.00 (Fig. 2A–B, Table 2). The next best-performing model was AdaBoost, with an AUC of 0.98 (95% CI: 0.97–0.99).

**Table 2:** Performance of ML algorithms in training cohort and validation cohort

**Cohort**	**ML Algorithms**		**AUC (95%CI)**		**Accuracy (95%CI)**		**Sensitivity (95%CI)**		**Specificity (95%CI)**		**PPV (95%CI)**	**NPV (95%CI)**	**F1 (95%CI)**
	XGBoost		1.00 (NaN-NaN)		1.0(1.00–1.00)		1.0(1.00–1.00)		1.0(1.00–1.00)		1.0(1.00–1.00)	1.0(1.00–1.00)	1.0(1.00–1.00)
	logistic		0.86 (0.82–0.90)		0.85(0.84–0.85)		0.74(0.72–0.75)		0.86(0.86–0.87)		0.49(0.47–0.50)	0.95(0.95–0.95)	0.59(0.58–0.60)
	LightGBM		1.00 (NaN-NaN)		1.0(1.00–1.00)		1.0(1.00–1.00)		1.0(1.00–1.00)		1.0(1.00–1.00)	1.0(1.00–1.00)	1.0(1.00–1.00)
	RandomForest		1.00 (NaN-NaN)		1.0(1.00–1.00)		1.0(1.00–1.00)		1.0(1.00–1.00)		0.99(0.98–1.00)	1.0(1.00–1.00)	0.99(0.99–1.00)
Training	AdaBoost		0.98 (0.97–0.99)		0.9(0.89–0.91)		0.98(0.97–0.99)		0.89(0.88–0.90)		0.61(0.58–0.64)	1.0(0.99–1.00)	0.75(0.73–0.77)
	GBDT		1.00 (NaN-NaN)		1.0(0.99–1.00)		0.99(0.98–0.99)		1.0(0.99–1.00)		0.99(0.97–1.01)	1.0(1.00–1.00)	0.99(0.98–1.00)
	GNB		0.86 (0.82–0.91)		0.85(0.83–0.87)		0.71(0.69–0.73)		0.88(0.85–0.90)		0.51(0.47–0.55)	0.95(0.94–0.95)	0.59(0.57–0.61)
	SVM		0.93 (0.90–0.96)		0.9(0.90–0.90)		0.86(0.85–0.87)		0.91(0.90–0.91)		0.62(0.61–0.62)	0.97(0.97–0.97)	0.72(0.71–0.72)
	XGBoost		0.88 (0.78–0.98)		0.88(0.86–0.90)		0.32(0.24–0.41)		0.98(0.97–0.99)		0.73(0.60–0.85)	0.89(0.88–0.91)	0.44(0.34–0.53)
Validation	logistic		0.85 (0.72–0.98)		0.83(0.81–0.85)		0.68(0.58–0.78)		0.85(0.83–0.88)		0.45(0.41–0.49)	0.94(0.92–0.96)	0.53(0.48–0.59)
	LightGBM		0.90 (0.81–0.99)		0.88(0.87–0.89)		0.57(0.47–0.68)		0.93(0.91–0.96)		0.62(0.54–0.70)	0.93(0.91–0.94)	0.58(0.51–0.66)
	RandomForest		0.87 (0.75–0.98)		0.88(0.86–0.90)		0.23(0.16–0.29)		1.0(0.99–1.00)		0.93(0.86–1.00)	0.88(0.87–0.89)	0.35(0.27–0.43)
	AdaBoost		0.83 (0.67–0.97)		0.84(0.82–0.86)		0.71(0.62–0.80)		0.86(0.84–0.89)		0.48(0.43–0.53)	0.94(0.93–0.96)	0.57(0.51–0.62)
	GBDT		0.89 (0.79–0.99)		0.89(0.87–0.91)		0.61(0.53–0.70)		0.94(0.92–0.96)		0.66(0.59–0.72)	0.93(0.92–0.95)	0.62(0.57–0.68)
	GNB		0.86 (0.75–0.98)		0.84(0.81–0.87)		0.68(0.58–0.78)		0.87(0.83–0.90)		0.49(0.42–0.56)	0.94(0.92–0.96)	0.56(0.50–0.61)
	SVM		0.86 (0.73–0.98)		0.8(0.79–0.81)		0.75(0.69–0.82)		0.81(0.80–0.83)		0.41(0.39–0.43)	0.95(0.94–0.96)	0.53(0.50–0.56)

CI, Confidence interval; PPV, positive predictive value; NPV, negative predictive value; ML, machine learning; GBDT, Gradient Boosting Decision Tree; LightGBM, Light Gradient Boosting Machine; AdaBoost, Adaptive Boosting; XGBoost, eXtreme Gradient Boosting; GNB, Gaussian Naive Bayes s; SVM, Support Vector Machine

**Fig. 3:**
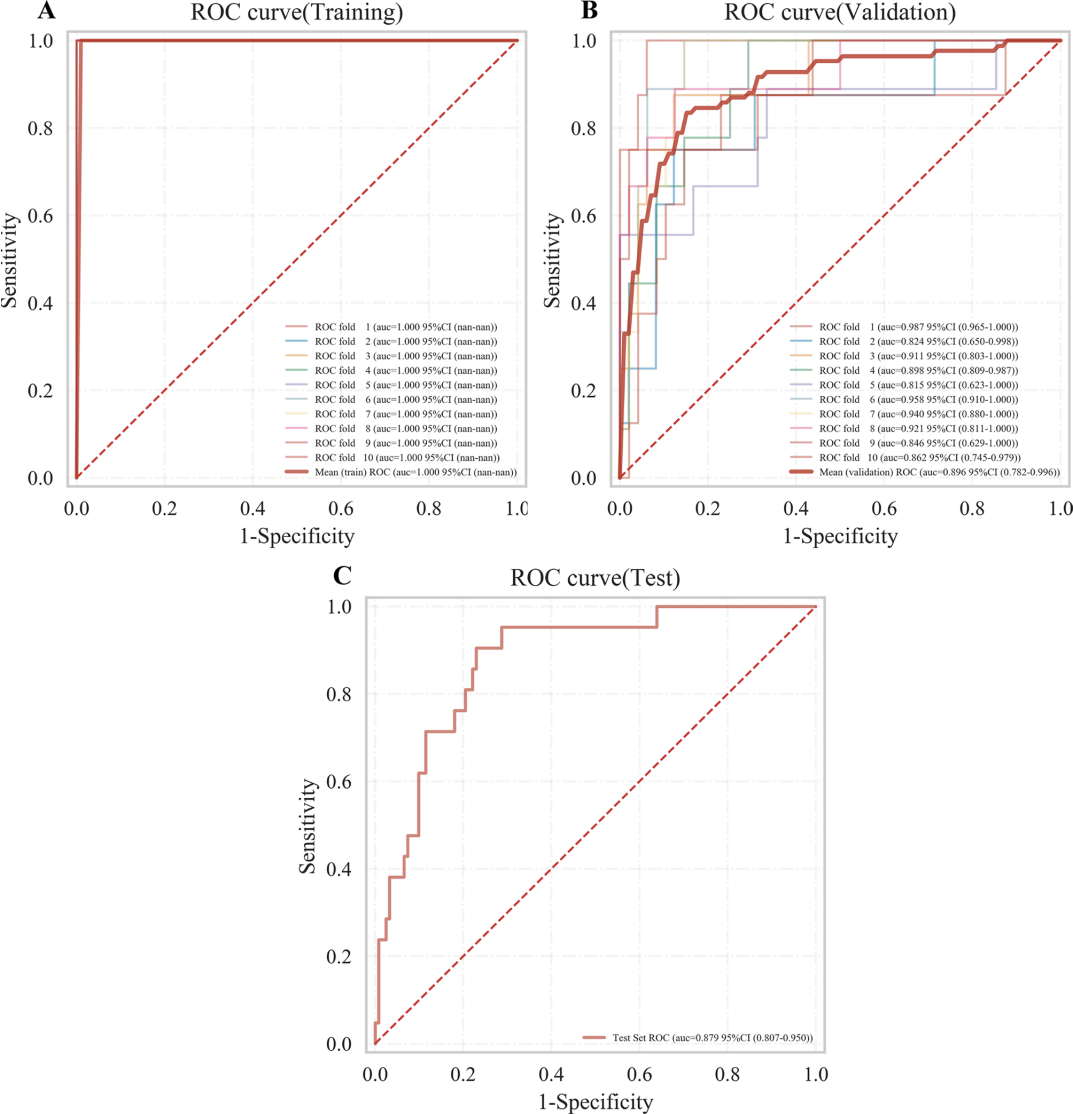
ROC curves of LightGBM algorithm. **A**. ROC curve of the training set; **B**. ROC curve of the validation set; **C**. ROC curve of the test set. ROC, receiver operating characteristics; AUC, area under ROC; LightGBM, light gradient boosting machine

However, in the validation cohort, the performance of XGBoost, RF, and GBDT declined considerably (Fig. 3C–D, Table 2), yielding AUCs of 0.88 (95% CI: 0.78–0.98), 0.87 (95% CI: 0.75–0.98), and 0.89 (95% CI: 0.79–0.99), respectively. In contrast, LightGBM outperformed the other models, achieving an AUC of 0.90 (95% CI: 0.81–0.99) with highest PR-AUC of 0.70 (95% CI: 0.65–0.76). Its corresponding accuracy, sensitivity, specificity, PPV, and NPV were 0.88 (95% CI: 0.87–0.89), 0.57 (95% CI: 0.47–0.68), 0.93 (95% CI: 0.91–0.96), 0.62 (95% CI: 0.54–0.70), and 0.93 (95% CI: 0.91–0.94), respectively. Based on this comprehensive evaluation, LightGBM was selected as the final algorithm for model construction.

### Performance of the LightGBM model

The LightGBM model was retrained on the training and validation cohorts, assessed on the test cohort. The LightGBM model achieved an AUC of 0.97 (95% CI: 0.96–0.98) on the training cohort and 0.89 (95% CI: 0.83–0.95) on the validation cohort. In the test cohort, the AUC remained consistent at 0.88 (Fig. 3A–C).

**Fig. 4:**
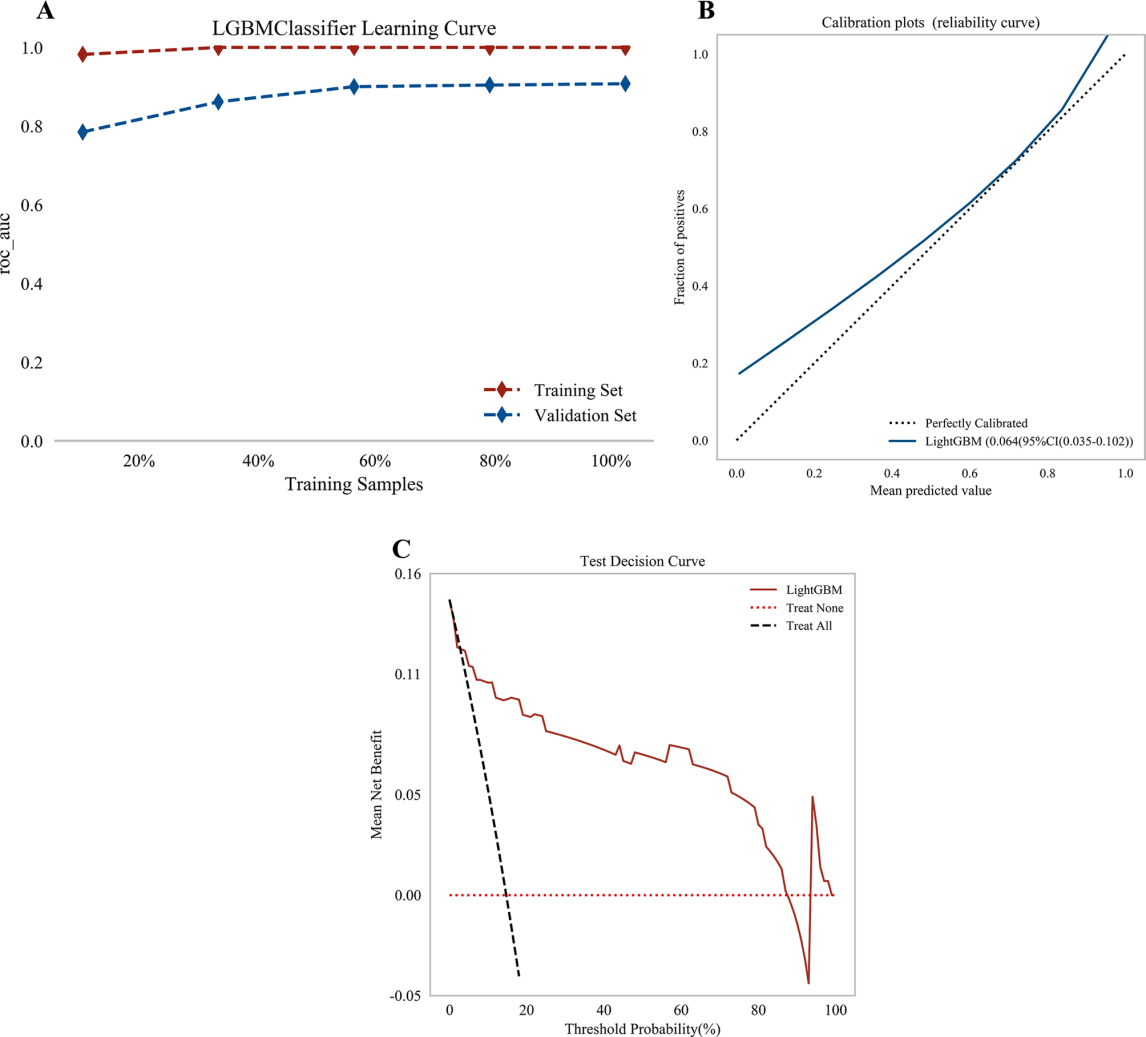
Learning curve, calibration curve and DCA curve of the model. **A**. Learning curves of the model; **B**. Calibration curve of the model; **C**. DCA curve of the model. LightGBM, light gradient boosting Machine; DCA, decision curve analysis

The learning curve (Fig. 4A) demonstrates that as the sample size increases, the model’s predictive performance on the validation set improves progressively. This trend suggests that the LightGBM model exhibits good generalization ability and avoids both underfitting and overfitting. Moreover, it underscores the importance of large sample sizes in achieving stable predictions. The calibration curve (Fig. 4B) indicates that the predicted probabilities closely align with the observed outcomes, with an expected calibration error of 0.064 (95% CI: 0.035–0.102). The DCA (Fig. 4C) further illustrates that the model yields positive net clinical benefit across a wide range of threshold probabilities (0%–85%).

### Model interpretation based on SHAP

**Fig. 5:**
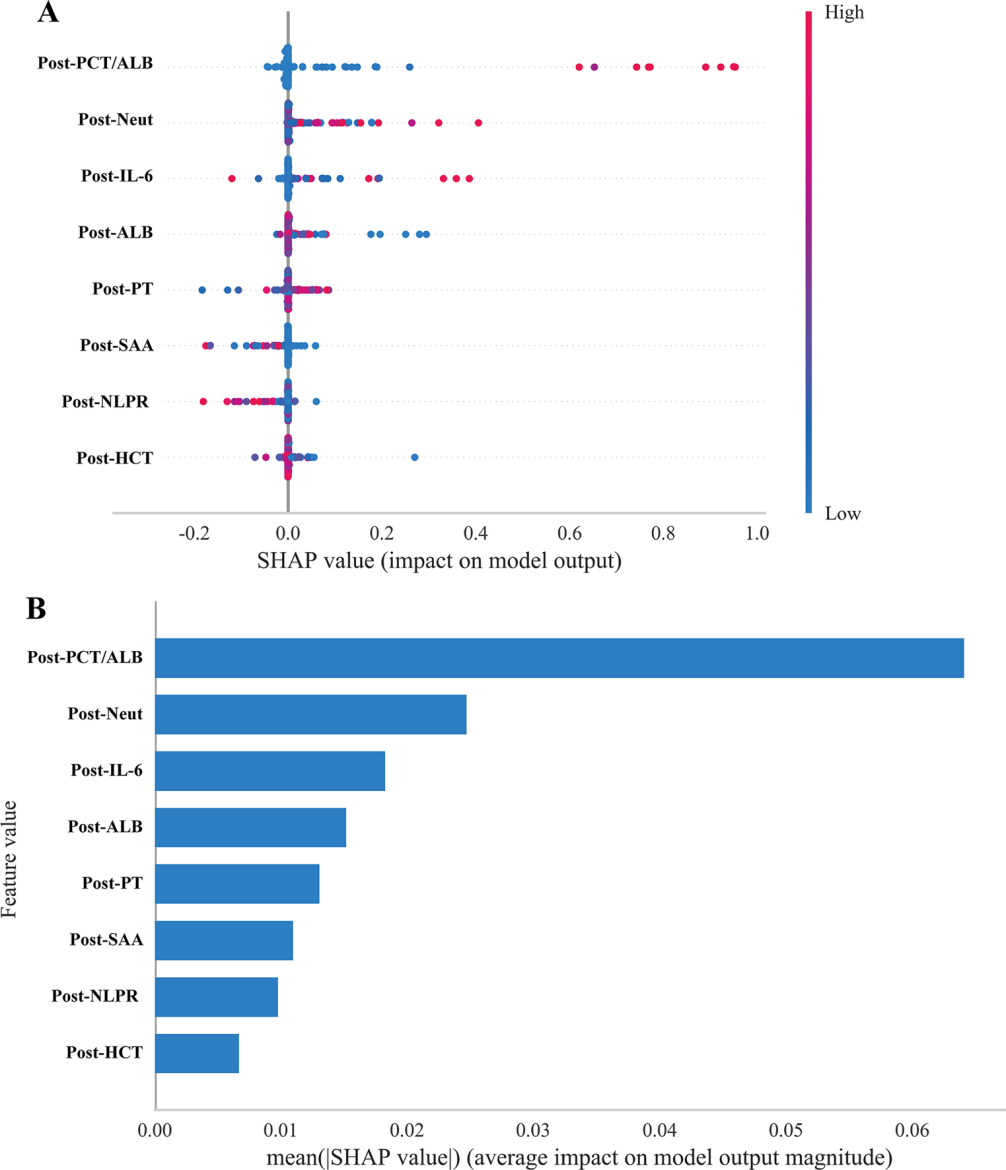
Visualization of variable Importance. **A**. shap summary plot; **B**. shap waterfall plot. shap, shapley additive Explanations; POST-IL-6: postoperative interleukin-6; POST-SAA: postoperative serum amyloid a; POST-PCT/ALB: postoperative Procalcitonin/Albumin; POST-NLPR: postoperative neutrophil to lymphocyte prognostic Ratio; POST-PT: postoperative prothrombin Time; POST-ALB: postoperative Albumin; POST-HCT: postoperative Hematocrit; POST-Neut: postoperative neutrophil

To enhance the interpretability of the model and facilitate clinical application by avoiding the “black box” effect, we employed SHAP analysis to visualize the contribution of each variable to the model’s predictions. The results indicated that postoperative PCT/ALB, neutrophil, IL-6, ALB, and PT were the top five predictors of urosepsis (Fig. 5A–B). Higher levels of postoperative PCT/ALB, neutrophil, IL-6, ALB, and PT were associated with an increased likelihood of urosepsis. We further evaluated the predictive performance of individual variables, which yielded results consistent with the SHAP analysis. Postoperative PCT/ALB, IL-6, and the HCT ratio emerged as the most predictive variables, with AUCs of 0.85, 0.78, and 0.73, respectively (Supplementary Figure 2). Additionally, postoperative PT and ALB each achieved AUCs of 0.70.

**Fig. 6:**
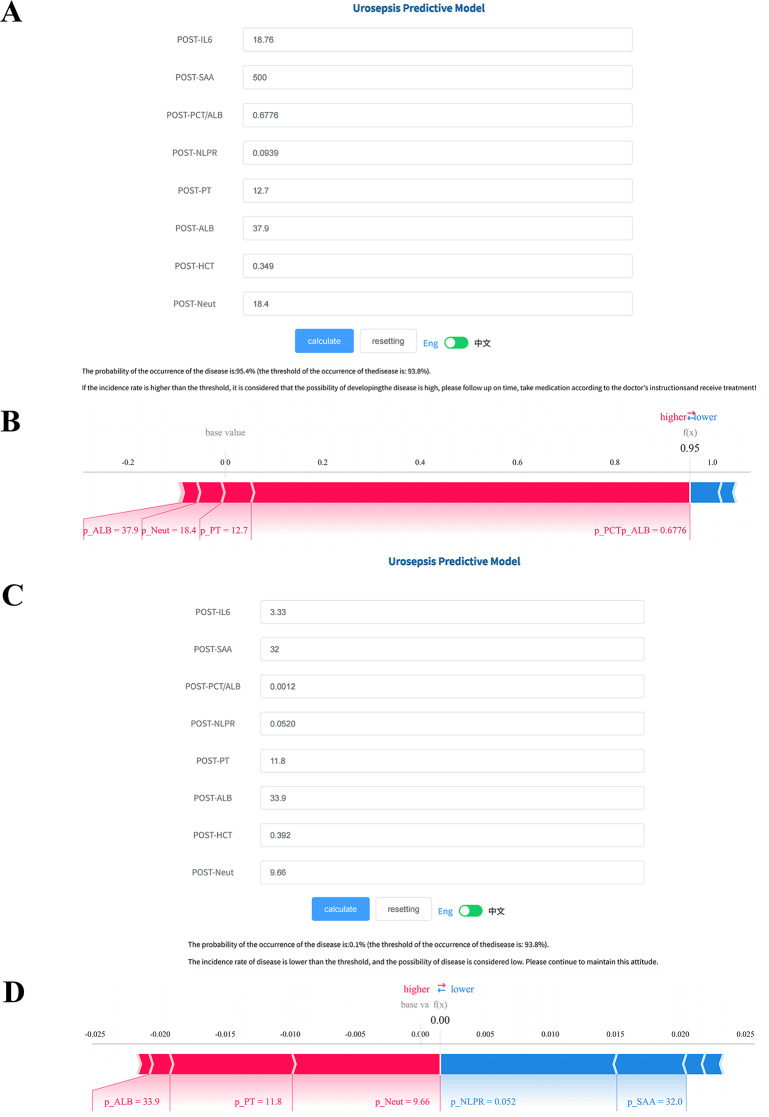
The application of online app. **A**. The web page indicated a high risk of developing urosepsis after patient 1 input the variables. **B**. The shap force plot of patient 1 visualized the contribution of relevant variables to the predicted urosepsis. **C**. The web page indicated a low risk of urosepsis after patient 2 input the variables. **D**. The shap force plot of patient 2 visualized the contribution of relevant variables to the predicted non-urosepsis. shap, shapley additive Explanations; POST-IL-6: postoperative interleukin-6; POST-SAA: postoperative serum amyloid a; POST-PCT/ALB: postoperative Procalcitonin/Albumin; POST-NLPR: postoperative neutrophil to lymphocyte prognostic Ratio; POST-PT: postoperative prothrombin Time; POST-ALB: postoperative Albumin; POST-HCT: postoperative Hematocrit; POST-Neut: postoperative neutrophil

To enhance accessibility for clinicians and patients at other centers, we developed the final model into a publicly accessible application (https://www.xsmartanalysis.com/model/list/predict/model/html?mid=29648&symbol=71lphm763138mj895nd8). Figure 6 illustrates the application interface, where users can input test results for the predictive variables to generate the estimated probability of urosepsis.

For Patient 1, the postoperative values of IL-6, SAA, PCT/ALB, NLPR, PT, ALB, HCT, and Neut were 18.76 pg/mL, 500 mg/L, 0.6876, 0.0939, 12.7 s, 37.9 mg/L, 0.349 g/L and 18.4 × 10^9^/L, respectively (Fig. 6A). The model predicted a urosepsis probability of 95.4%, exceeding the predefined threshold, thus categorizing the patient as high risk. This prediction aligned with the actual clinical outcome. The SHAP force plot visualized the contributions of individual variables to this prediction (Fig. 6B).

For Patient 2, the corresponding postoperative values were 3.33 pg/mL, 32 **mg/L**, 0.0012, 0.0520, 11.8 s, 33.9 mg/L, 0.392 g/L and 9.66 × 10^9^/L (Fig. 6C). The model predicted a urosepsis probability of 0.1%, which was below the risk threshold, indicating a low likelihood of disease—consistent with the patient’s clinical outcome. The SHAP force plot again illustrated the individual contributions to the prediction (Fig. 6D).

## Discussion

Urosepsis is a severe and potentially life-threatening complication following surgical treatment of upper urinary tract stones. Identifying key predictive factors is essential for guiding effective therapeutic strategies. Traditional risk assessment methods often depend on clinical experience and single-variable indicators, which may lack sufficient predictive accuracy.

In this study, we developed a predictive tool for urosepsis using multiple ML algorithms and comprehensive patient data collected from our hospital. We analyzed data from 7464 patients who underwent PCNL, RIRS, or URL. Following careful feature selection, eight clinical variables were ultimately included in model development. Eight ML algorithms were applied for model construction and validation. Among them, the LightGBM model demonstrated the highest predictive performance, with AUCs of 1.0, 0.90, and 0.88 in the training, validation, and test cohorts, respectively. The learning curve, calibration plot, and decision curve analysis indicated a good model fit, strong calibration, and substantial clinical benefit. Postoperative levels of PCT/ALB, neutrophil, IL-6, ALB, and PT were identified as key predictors using the SHAP algorithm. Furthermore, to evaluate the predictive power of individual variables, the AUCs of postoperative PCT/ALB, IL-6, and the HCT were 0.85, 0.78, and 0.73, respectively. An online platform based on the LightGBM model was also developed, enabling clinicians and patients to assess urosepsis risk and support timely intervention.

We carefully selected several parameters for model construction, including postoperative IL-6, SAA, PCT/ALB, NLPR, PT, ALB, HCT, and neutrophil. SAA is an acute-phase protein that increases sharply in plasma during inflammatory responses, such as those seen in sepsis. Its dynamic fluctuations are associated with patient prognosis, and combined measurement with other biomarkers such as CRP and PCT facilitates early identification of sepsis [23]. Consistent with previous studies, PCT remains a key biomarker for early diagnosis. Evidence suggests that elevated PCT levels can be detected early in patients with sepsis [24]. However, the diagnostic performance of PCT varies across studies, with reported AUC values ranging from 0.7 to 0.9. These discrepancies may be attributed to differences in patient cohorts and potential biases inherent in observational studies [25, 26]. Notably, studies specifically focused on the diagnostic utility of PCT in urosepsis remain limited. In our study, PCT demonstrated an AUC of 0.81 for predicting urosepsis and was identified as the most important predictor by SHAP analysis. IL-6 is rapidly induced during the acute phase of infection or tissue injury and contributes to host defense by stimulating the production of acute-phase proteins and enhancing immune responses [27]. Combined detection of IL-6 and IL-10 has shown superior diagnostic accuracy for bacteremia and severe infections compared to PCT, particularly in pediatric patients with hematologic malignancies [28]. Zhang et al. reported that IL-6 achieved an AUC of 0.679 in differentiating Gram-positive from Gram-negative sepsis [29]. In a rat model of urosepsis, Cao et al. found that elevated IL-6 levels were significantly associated with multiple organ dysfunction [30].

Patients with sepsis commonly exhibit elevated WBC, neutrophils, and NLPR, along with lymphopenia, all of which are correlated with the severity of infection [31]. Neutrophil dysfunction, characterized by diminished elastase release and reduced reactive oxygen species production, is a hallmark of sepsis. Among neutrophil surface markers, CD64 has emerged as a highly promising biomarker, demonstrating significant elevation in sepsis and an AUC exceeding 0.9 in several studies [32, 33]. HCT plays a critical role in determining blood viscosity and may contribute to sepsis-related organ dysfunction through microvascular obstruction. Although dynamic changes in HCT levels have been observed in patients with sepsis, its diagnostic value remains under investigation [34]. Cao et al. reported that serum albumin (ALB) levels were significantly lower in the sepsis group compared to the non-sepsis group, with an AUC of approximately 0.73 for sepsis diagnosis, consistent with our findings [35]. Sepsis is frequently associated with coagulation dysfunction, and prolonged PT is one of its characteristic manifestations. Prolonged PT indicates the consumption of coagulation factors in patients with sepsis, which results from excessive activation [36].

Interestingly, our differential analysis revealed that multiple preoperative laboratory parameters had already exhibited abnormalities. A high preoperative Systemic Immune-Inflammation Index (SII) has been identified as an independent risk factor for postoperative sepsis in patients undergoing surgery for intestinal obstruction [37]. Nedbal et al. also reported a positive association between postoperative sepsis and preoperative urine culture results [38]. However, Sui et al. argued that abnormal preoperative laboratory tests are not significant independent predictors of postoperative complications in low-risk ambulatory urological procedures, although this study did not include stone-related surgeries [39]. These findings suggest that patients who develop urosepsis may already exhibit preoperative pathophysiological changes, highlighting the potential for early prediction of urosepsis before surgery.

The integration of ML-based prediction models into clinical workflows offers several advantages. First, ML models can efficiently process large volumes of multidimensional clinical data, offering real-time and dynamic risk assessments superior to those provided by traditional statistical methods. Second, by incorporating key postoperative parameters, our model facilitates personalized decision-making, enabling clinicians to identify patients at high risk for urosepsis prior to clinical deterioration. Third, interpretability techniques such as SHAP enhance transparency by linking model predictions to biologically meaningful variables, thereby increasing clinician trust and model reliability. To our knowledge, this study constitutes the largest real-world investigation with the largest sample size to date for predicting urosepsis. Prior studies were limited by small sample sizes, which increased susceptibility to bias. Moreover, the integration of the SHAP algorithm and a user-friendly online platform supports greater acceptance among clinicians and patients.

Despite its promising findings, this study has several limitations. First, as a retrospective analysis, it is subject to selection bias; thus, external validation using prospective, multi-center datasets is necessary to improve the generalizability of our results. Second, although the model incorporates essential laboratory variables, it lacks advanced biomolecular markers, which could enhance predictive accuracy. Imaging also plays a vital role in the early diagnosis and risk stratification of urosepsis by revealing anatomical abnormalities and complications [40]. Future studies should integrate radiomics and clinical data to improve prediction accuracy. Third, based on the strong performance of the LightGBM model, we developed an online platform for clinical use. However, variations in clinical feature measurements across institutions may affect the model’s predictive performance. Efforts should focus on standardizing clinical parameters based on reference ranges. Fourth, while LightGBM performed well, the use of deep learning models incorporating longitudinal patient data may further improve risk predictions. An additional limitation pertains to the interpretability of the model. Although the SHAP algorithm provided transparent insights into the contribution of each predictor, we did not validate the robustness of SHAP values. This means the reported variable importance rankings may be susceptible to sample-specific randomness, and their generalizability to other datasets cannot be fully confirmed without further validation. Future research should address this by incorporating stability testing of SHAP attributions. Notably, the final model relies exclusively on postoperative parameters, allowing for timely risk stratification. However, this also limits the model’s utility for preoperative risk assessment. Future investigations should explore integrating preoperative variables (e.g., preoperative urine culture results, anatomical factors) to develop a dual-stage prediction model for more comprehensive risk management. The last but not the least, although we included demographic and baseline clinical characteristics in the initial feature set, these variables were not retained in the final model due to non-significant group differences. However, adjusting for these factors may help control potential confounding and improve generalizability. Future studies could incorporate these variables as covariates in the model to validate their impact on predictive performance.

## Conclusions

This study successfully developed and validated a ML-based predictive model for postoperative urosepsis in patients undergoing surgery for upper urinary tract stones. LightGBM demonstrated the highest predictive performance, offering a highly accurate, interpretable, and clinically applicable tool for risk stratification. By enabling early identification of high-risk patients, the model has the potential to improve clinical decision-making. Future research should focus on external validation and the broader integration of ML-driven decision-support tools into routine clinical practice.

## Electronic supplementary material

Below is the link to the electronic supplementary material.

Supplementary Material 1

Supplementary Material 2

Supplementary Material 3

Supplementary Material 4

Supplementary Material 5

Supplementary Material 6

Supplementary Material 7

Supplementary Material 8

## Data Availability

The datasets generated and/or analyzed are not publicly available owing to ethical and legal causes. Nevertheless, they can be made available from the corresponding author on reasonable request.

## Abbreviations

PCNL: Percutaneous Nephrolithotomy
RIRS: Retrograde Intrarenal Surgery
URL: Ureteroscopic Lithotripsy
PCT: Procalcitonin
CRP: C-reactive Protein
AUC: Area Under the Receiver Operating Characteristic Curve
PR-AUC: Area Under the Precision-Recall Curve
ML: Machine Learning
SHAP: SHapley Additive exPlanations
ETL: Extract-Transform-Load
VTE: Venous Thromboembolism
ASA: American Society of Anesthesiologists
NLPR: neutrophil-lymphocyte-platelet ratio
LASSO: Least Absolute Shrinkage and Selection Operator
GBDT: Gradient Boosting Decision Tree
LightGBM: Light Gradient Boosting Machine
AdaBoost: Adaptive Boosting
XGBoost: eXtreme Gradient Boosting
GNB: Gaussian Naive Bayes
SVM: Support Vector Machine
ICU: Intensive Care Unit
SAA: Serum Amyloid A
IL-6: Interleukin-6
WBC: White Blood Cell
Neut: Neutrophils
HCT: Hematocrit
β2-MG: β2-microglobulin
ALB: Albumin
PPV: Positive Predictive Value
NPV: Negative Predictive Value
DCA: Decision Curve Analysis
SII: Systemic Immune-Inflammation Index
