# Supplementary material for: Leveraging laboratory biomarkers to predict urosepsis after upper urinary tract stone surgery: an explainable machine learning approach
Source: BMC Med Inform Decis Mak. 2025 Dec 20;26:27. doi: 10.1186/s12911-025-03314-y (PMC12838489; doi:10.1186/s12911-025-03314-y)
Supplement: Supplementary file 1 — Supplementary Material 1 [file 12911_2025_3314_MOESM1_ESM.pdf]

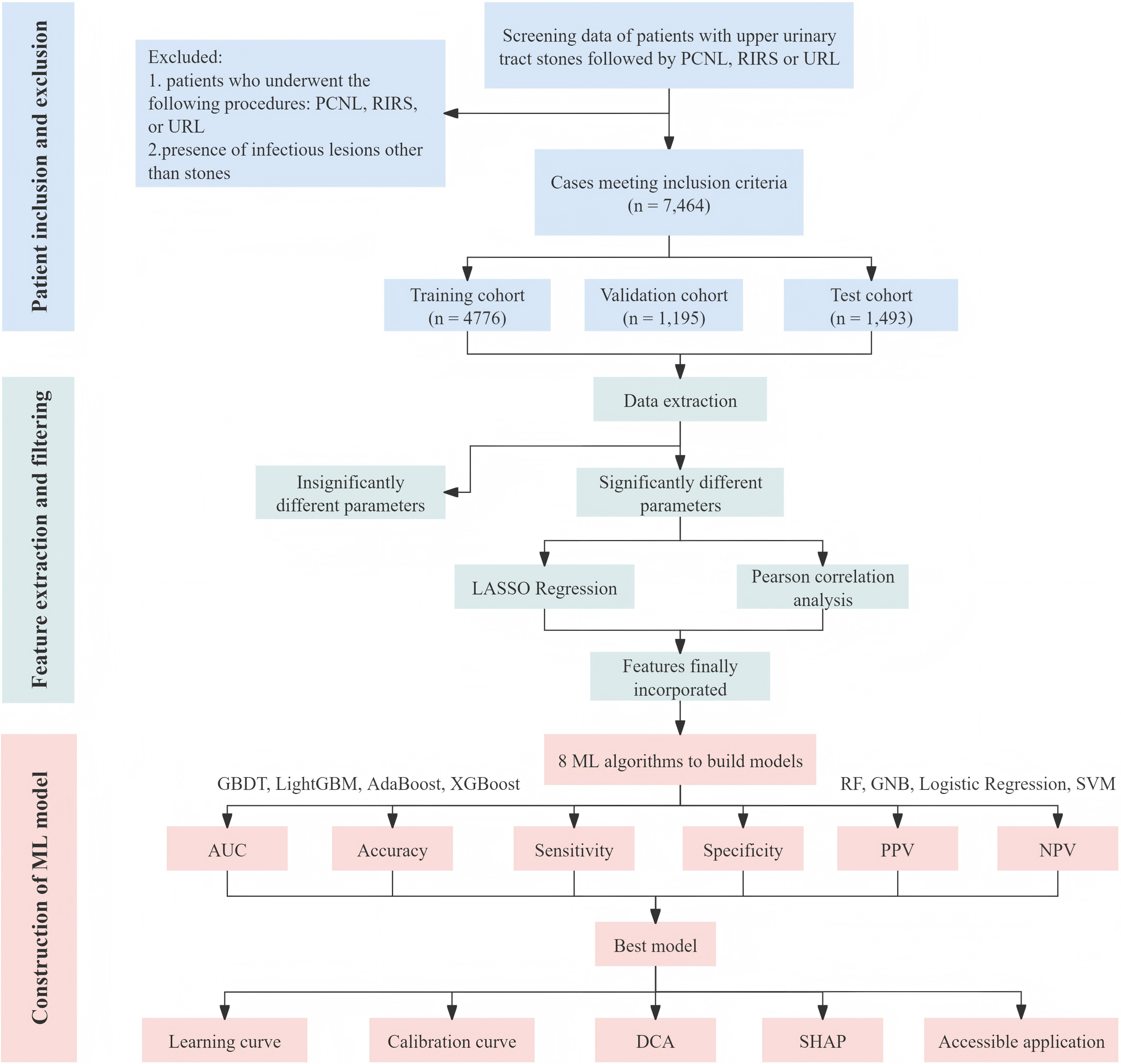

Patient inclusion and exclusion
Screening data of patients with upper urinary tract stones followed by PCNL, RIRS or URL
Excluded:
1. patients who underwent the following procedures: PCNL, RIRS, or URL
2.presence of infectious lesions other than stones
Cases meeting inclusion criteria (n = 7,464)
Training cohort (n = 4776)
Validation cohort (n = 1,195)
Test cohort (n = 1,493)
Feature extraction and filtering
Data extraction
Insignificantly different parameters
Significantly different parameters
LASSO Regression
Pearson correlation analysis
Features finally incorporated
Construction of ML model
8 ML algorithms to build models
GBDT, LightGBM, AdaBoost, XGBoost
RF, GNB, Logistic Regression, SVM
AUC
Accuracy
Sensitivity
Specificity
PPV
NPV
Best model
Learning curve
Calibration curve
DCA
SHAP
Accessible application
