# Supplementary figures and images for: Leveraging laboratory biomarkers to predict urosepsis after upper urinary tract stone surgery: an explainable machine learning approach
Source: BMC Med Inform Decis Mak. 2025 Dec 20;26:27. doi: 10.1186/s12911-025-03314-y (PMC12838489; doi:10.1186/s12911-025-03314-y)

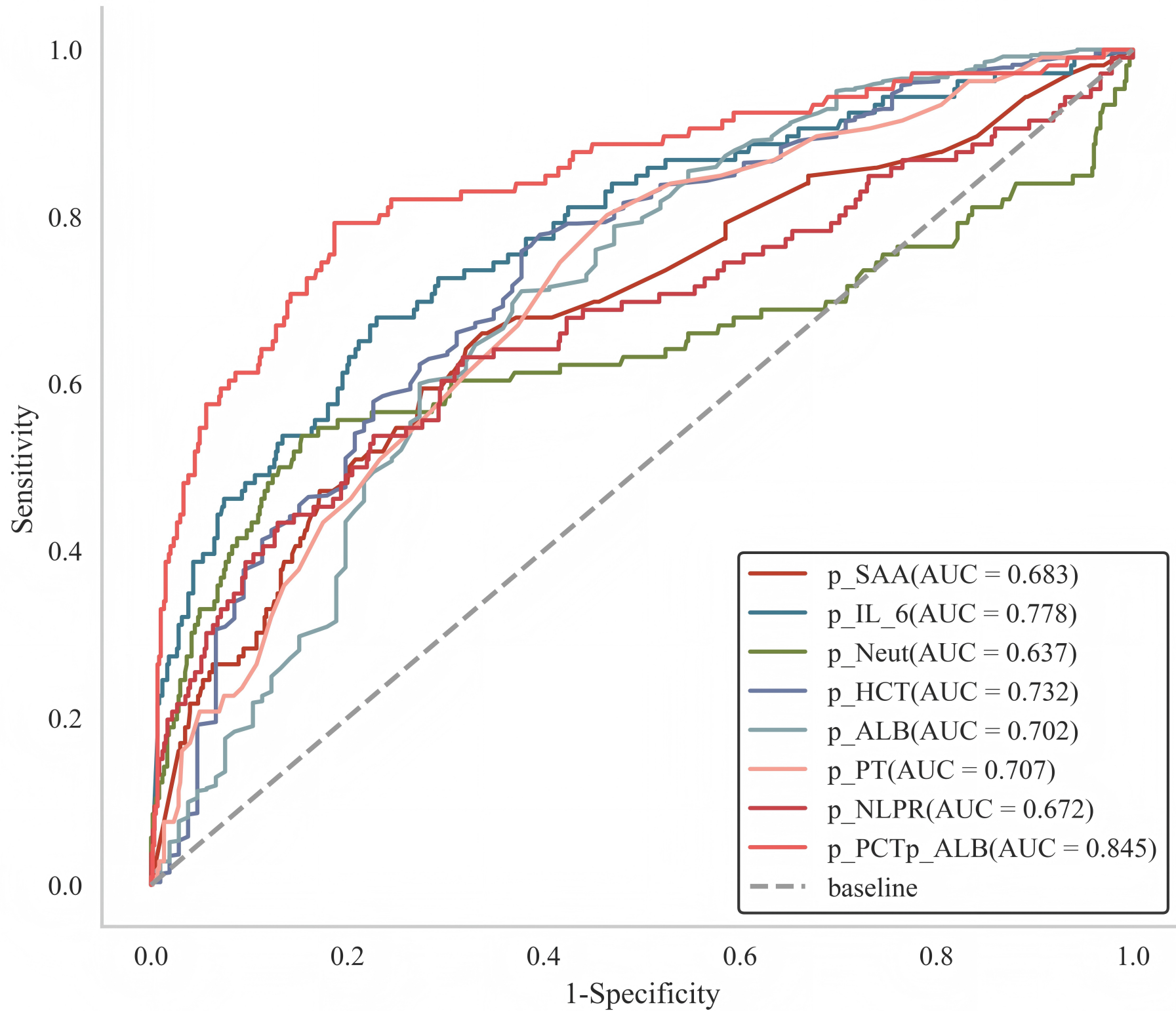

Supplement: Supplementary file 2 — Supplementary Material 2 [file 12911_2025_3314_MOESM2_ESM.pdf]
