# Supplementary material for: Leveraging laboratory biomarkers to predict urosepsis after upper urinary tract stone surgery: an explainable machine learning approach
Source: BMC Med Inform Decis Mak. 2025 Dec 20;26:27. doi: 10.1186/s12911-025-03314-y (PMC12838489; doi:10.1186/s12911-025-03314-y)
Supplement: Supplementary file 3 — Supplementary Material 3 [file 12911_2025_3314_MOESM3_ESM.pdf]

Supplementary Table 1. STROBE Statement—Checklist of items that should be included in reports of *cohort studies*

	Item No	Recommendation	Page No.	Relevant text from manuscript
**Title and abstract**	1	(*a*) Indicate the study's design with a commonly used term in the title or the abstract	1	Leveraging Laboratory Biomarkers to Predict Urosepsis After Upper Urinary Tract Stone Surgery: An Explainable Machine Learning Approach
		(*b*) Provide in the abstract an informative and balanced summary of what was done and what was found	3	116 out of 155 variables (74.84%) differed significantly between the urosepsis (n = 622, 8.33%) and non-urosepsis groups (n = 6,842, 91.67%). LASSO regression identified eight predictive variables: postoperative IL-6, SAA, PCT/ALB, NLPR, PT, ALB, HCT, and neutrophil. Light Gradient Boosting Machine achieved the best performance, with AUCs of 1.0, 0.90, and 0.88 for the training, validation, and test cohorts, respectively. A good model fit, strong calibration, and positive clinical utility were confirmed by the learning curve, calibration plot, and decision curve. Postoperative PCT/ALB, neutrophil, IL-6, ALB, and PT were identified as key predictors by the SHAP algorithm.
**Introduction**				
Background/rationale	2	Explain the scientific background and rationale for the investigation being reported	5-6	Urolithiasis is a common condition in urology, with a lifetime prevalence of 10–20% and an annual incidence of approximately 1% [1], particularly with upper urinary tract stones being more common [2]. With advances of minimally invasive techniques, procedures such as percutaneous nephrolithotomy (PCNL), retrograde intrarenal surgery (RIRS), and ureteroscopic lithotripsy (URL) have become the first-line treatments for upper urinary tract stones [3]. Minimally invasive techniques offer advantages such as minimal trauma, high stone clearance rates, and rapid postoperative recovery. However, they may lead to complications such as bleeding and infection [4]. Among these, urosepsis is the most severe complication and a common cause of perioperative mortality [5]. Urosepsis results from the invasion of microorganisms and their toxins into the bloodstream, leading to systemic inflammatory response syndrome and organ dysfunction, even death in severe cases [6]. Urosepsis can escalate to septic shock, with a mortality rate as high as 66%–80% [7].<br>Early identification and effective control of urosepsis are crucial for improving patient outcomes and survival rates. According to international guidelines for the management of sepsis and septic shock, antibiotics should be administered within the first hour of recognition [8]. Studies indicate that each one-hour delay in antibiotic administration

				increases the risk of septic shock by 1.8%, and overall mortality rises by 3-13% in patients with sepsis [9]. Blood culture is essential for the diagnosis of sepsis, especially in the neonatal field, where blood culture (including peripheral and umbilical cord blood) is considered the "gold standard" [10]. However, blood culture has notable limitations, including a long turnaround time (48-72 hours) and low sensitivity [11]. Emerging biomarkers offer potential for earlier identification of sepsis and may assist in identifying causative pathogens. To date, hundreds of sepsis-related biomarkers have been studied, including procalcitonin (PCT), C-reactive protein (CRP), lactate, and various cytokines, among which PCT and CRP are the most widely used in clinical practice [12]. In healthy individuals, PCT is secreted by neuroendocrine cells of the thyroid, but during infection, it can be released by a variety of cell types [13]. PCT levels rise within 2-4 hours after infection and have better diagnostic performance than other inflammatory markers [14]. Nonetheless, Dragoescu et al. found that the area under the receiver operating characteristic curve (AUC) of PCT for diagnosing urosepsis was only 0.743 [15]. The AUC of CRP for sepsis diagnosis is 0.77, which is comparable to the diagnostic performance of PCT [16]. Cytokines are significantly elevated in sepsis patients and participate in the regulation of inflammatory responses, but their diagnostic efficacy is inferior to that of PCT and CRP [17]. These limitations highlight the urgent need for more accurate and timely methods for the early diagnosis of urosepsis.
Objectives	3	State specific objectives, including any prespecified hypotheses	6	To address these challenges, we developed a Machine learning (ML) model to predict urosepsis based on large-scale clinical data from patients with upper urinary tract stones. To enhance model interpretability and support clinical decision-making, we applied the SHapley Additive exPlanations (SHAP) algorithm to explain the model's predictions.
**Methods**				
Study design	4	Present key elements of study design early in the paper	7-8	This real-world, retrospective study was conducted at the Sixth Affiliated Hospital of Guangxi Medical University, China (The First People's Hospital of Yulin). Clinical data were collected from the electronic medical records of 7,464 patients between January 2018 and June 2023.<br>Initially, 20% of patients were randomly sampled from the entire cohort to serve as the test cohort, while the remaining 80% were randomly split into the training cohort and validation cohort at a ratio of 8:2. Feature selection was exclusively performed within the

				training cohort.<br>The test set was strictly held out and not involved in any step of feature selection or model training.
Setting	5	Describe the setting, locations, and relevant dates, including periods of recruitment, exposure, follow-up, and data collection	7	This real-world, retrospective study was conducted at the Sixth Affiliated Hospital of Guangxi Medical University, China (The First People's Hospital of Yulin). Clinical data were collected from the electronic medical records of 7,464 patients between January 2018 and June 2023.
Participants	6	(*a*) Give the eligibility criteria, and the sources and methods of selection of participants. Describe methods of follow-up	7	Inclusion criteria were as follows: (1) age ≥ 18 years; (2) diagnosis of upper urinary tract stones confirmed by ultrasound or computed tomography, followed by minimally invasive surgery (PCNL, RIRS or URL); (3) availability of complete clinical records; (4) diagnosis of urosepsis (experimental group) or absence of urosepsis (control group). Exclusion criteria were: (1) simultaneous performance of more than one surgical procedure; (2) presence of infectious lesions unrelated to urolithiasis. All urosepsis cases were diagnosed within 48 hours after surgery.
		(*b*) For matched studies, give matching criteria and number of exposed and unexposed	-	Not applicable
Variables	7	Clearly define all outcomes, exposures, predictors, potential confounders, and effect modifiers. Give diagnostic criteria, if applicable	7-8	Multimodal clinical parameters of eligible participants before and after operation were extracted using extract-transform-load (ETL) tools. Clinical variables recorded for risk factor analysis and model development included age, gender, height, weight, history of hypertension and diabetes, and imaging reports (ultrasound and non-contrast computed tomography). Barthel Index scores, venous thromboembolism (VTE) risk assessments, Morse Fall Scale scores, information on whether patients had diabetes or hypertension, and the American Society of Anesthesiologists (ASA) physical status classification were also collected. In addition, laboratory test data obtained immediately before and after surgery were extracted.<br>In addition, we extracted laboratory test data obtained within 24 hours before surgery (preoperative laboratory data) and within 6 hours after surgery (postoperative laboratory data), distinguished by adding the prefixes "p-". Given the diagnostic value of systemic immune-inflammatory biomarkers in sepsis, we analyzed haematological biomarkers (e.g., white blood cell count, neutrophil count, hematocrit), biochemical biomarkers (e.g., albumin, β2-microglobulin, cystatin C), inflammatory biomarkers (e.g., PCT, CRP, IL-6, SAA), and immune-inflammatory ratios (e.g., neutrophil-to-lymphocyte ratio, platelet-to-

				lymphocyte ratio).<br>In total, 155 clinical multimodal features were included in this study.
Data sources/ measurement	8*	For each variable of interest, give sources of data and details of methods of assessment (measurement). Describe comparability of assessment methods if there is more than one group	8	Multimodal clinical parameters of eligible participants before and after operation were extracted using extract-transform-load (ETL) tools. Clinical variables recorded for risk factor analysis and model development included age, gender, height, weight, history of hypertension and diabetes, and imaging reports (ultrasound and non-contrast computed tomography). Barthel Index scores, venous thromboembolism (VTE) risk assessments, Morse Fall Scale scores, information on whether patients had diabetes or hypertension, and the American Society of Anesthesiologists (ASA) physical status classification were also collected. In addition, laboratory test data obtained immediately before and after surgery were extracted.<br>In addition, we extracted laboratory test data obtained within 24 hours before surgery (preoperative laboratory data) and within 6 hours after surgery (postoperative laboratory data), distinguished by adding the prefixes "p-". Given the diagnostic value of systemic immune-inflammatory biomarkers in sepsis, we analyzed haematological biomarkers (e.g., white blood cell count, neutrophil count, hematocrit), biochemical biomarkers (e.g., albumin, β2-microglobulin, cystatin C), inflammatory biomarkers (e.g., PCT, CRP, IL-6, SAA), and immune-inflammatory ratios (e.g., neutrophil-to-lymphocyte ratio, platelet-to-lymphocyte ratio).
Bias	9	Describe any efforts to address potential sources of bias	8-9	We analyzed the distribution of 155 clinical multimodal features between the experimental (urosepsis) and control (non-urosepsis) groups in the training cohort. For normally distributed continuous variables, Student's t-test was employed to compare intergroup differences. For non-normally distributed continuous variables, the Mann-Whitney U test was utilized. Categorical variables were compared via the chi-square test. Only variables with statistically significant differences ($P < 0.05$) were retained for subsequent analysis.<br>Next, we applied Least Absolute Shrinkage and Selection Operator (LASSO) regression with 10-fold cross-validation to identify parameters with non-zero coefficients. To assess multicollinearity among the selected features, Pearson correlation analysis was conducted, and features with an absolute correlation coefficient greater than 0.7 with any other feature were excluded.
Study size	10	Explain how the study size was arrived at	7	This real-world, retrospective study was conducted at the Sixth Affiliated Hospital of

				Guangxi Medical University, China (The First People's Hospital of Yulin). Clinical data were collected from the electronic medical records of 7,464 patients between January 2018 and June 2023.
Quantitative variables	11	Explain how quantitative variables were handled in the analyses. If applicable, describe which groupings were chosen and why	10, 8	Continuous variables were summarized as mean ± standard deviation for normally distributed data, or as median and interquartile range for non-normally distributed data. Student's t-test was used for normally distributed continuous variables, while the Mann-Whitney U test was applied for non-normally distributed ones.<br>We analyzed the distribution of 155 clinical multimodal features between the experimental (urosepsis) and control (non-urosepsis) groups in the training cohort.
Statistical methods	12	(*a*) Describe all statistical methods, including those used to control for confounding	10,	All data analyses were performed using Python software (version 3.8) and R (version 4.2.3). Continuous variables were summarized as mean ± standard deviation for normally distributed data, or as median and interquartile range for non-normally distributed data. Student's t-test was used for normally distributed continuous variables, while the Mann-Whitney U test was applied for non-normally distributed ones. Diagnostic metrics, including sensitivity, specificity, PPV, and NPV, were derived from the confusion matrix. A two-tailed P-value of less than 0.05 was considered statistically significant.
		(*b*) Describe any methods used to examine subgroups and interactions	-	Not applicable
		(*c*) Explain how missing data were addressed	7	Inclusion criteria were as follows: (1) age ≥ 18 years; (2) diagnosis of upper urinary tract stones confirmed by ultrasound or computed tomography, followed by minimally invasive surgery (PCNL, RIRS or URL); (3) availability of complete clinical records; (4) diagnosis of urosepsis (experimental group) or absence of urosepsis (control group).
		(*d*) If applicable, explain how loss to follow-up was addressed	7	All urosepsis cases were diagnosed within 48 hours after surgery.
		(*e*) Describe any sensitivity analyses	10	Learning curves were generated to illustrate changes in model performance with increasing sample size, allowing for the detection of potential underfitting or overfitting. Calibration plots, based on the Hosmer-Lemeshow test, were used to assess the agreement between predicted probabilities and observed outcomes. To address the interpretability challenge commonly associated with ML models, the SHAP algorithm was employed to quantify and visualize the contribution of each feature to model predictions, thereby enhancing transparency and aiding clinical interpretation.

Results

Participants	13*	(a) Report numbers of individuals at each stage of study—eg numbers potentially eligible, examined for eligibility, confirmed eligible, included in the study, completing follow-up, and analysed	11	Of the total study population, 4,486 (60.1%) were male and 2,978 (39.9%) were female. Notably, females were more prevalent in the urosepsis group ($P < 0.001$, Table 1). The urosepsis group also had a significantly higher proportion of patients undergoing percutaneous nephrolithotomy (57.88% vs. 38.53%, $P < 0.001$) and more frequent endoscopic procedures ($P < 0.001$). No significant differences were observed in the prevalence of diabetes or hypertension between the two groups ($P > 0.05$). Out of the 155 clinical features analyzed, 116 parameters (74.84%) showed significant differences between the groups ($P < 0.05$). We also collected several clinical scores, including the Barthel Index, VTE, Morse, and ASA scores. In the urosepsis group, VTE and ASA scores were higher, while the Barthel Index was lower ($P < 0.05$), with no significant difference in the Morse score ($P > 0.05$).
		(b) Give reasons for non-participation at each stage	7	Exclusion criteria were: (1) simultaneous performance of more than one surgical procedure; (2) presence of infectious lesions unrelated to urolithiasis. All urosepsis cases were diagnosed within 48 hours after surgery.
		(c) Consider use of a flow diagram	11	The study flowchart is shown in Supplementary Figure 1.
Descriptive data	14*	(a) Give characteristics of study participants (eg demographic, clinical, social) and information on exposures and potential confounders	11	Of the total study population, 4,486 (60.1%) were male and 2,978 (39.9%) were female. Notably, females were more prevalent in the urosepsis group ($P < 0.001$, Table 1). The urosepsis group also had a significantly higher proportion of patients undergoing percutaneous nephrolithotomy (57.88% vs. 38.53%, $P < 0.001$) and more frequent endoscopic procedures ($P < 0.001$). No significant differences were observed in the prevalence of diabetes or hypertension between the two groups ($P > 0.05$). Out of the 155 clinical features analyzed, 116 parameters (74.84%) showed significant differences between the groups ($P < 0.05$). We also collected several clinical scores, including the Barthel Index, VTE, Morse, and ASA scores. In the urosepsis group, VTE and ASA scores were higher, while the Barthel Index was lower ($P < 0.05$), with no significant difference in the Morse score ($P > 0.05$).
		(b) Indicate number of participants with missing data for each variable of interest	-	-
		(c) Summarise follow-up time (eg, average and total amount)	7	All urosepsis cases were diagnosed within 48 hours after surgery.

Outcome data	15*	Report numbers of outcome events or summary measures over time	11	Patients were divided into the experimental group (urosepsis, n = 622) and the control group (non-urosepsis, n = 6,842). Among the 622 patients who developed urosepsis, 527 (84.7%) stabilized after receiving appropriate treatment, including fluid resuscitation, anti-infective therapy, and administration of norepinephrine. 95 patients (15.3%) were transferred to the Intensive Care Unit for further treatment, and 3 patients (0.5%) died. The remaining patients showed improvement and were discharged.
Main results	16	(*a*) Give unadjusted estimates and, if applicable, confounder-adjusted estimates and their precision (eg, 95% confidence interval). Make clear which confounders were adjusted for and why they were included	13-14	Following 10-fold cross-validation, LightGBM, RF, GBDT and XGBoost demonstrated the highest performance in the training cohort, each achieving an AUC of 1.00. Furthermore, their accuracy, sensitivity, specificity, PPV, and NPV and PR-AUC all nearly reached 1.00 (Figure 2A-B, Table 2). The next best-performing model was AdaBoost, with an AUC of 0.98 (95% CI: 0.97-0.99).<br>However, in the validation cohort, the performance of XGBoost, RF, and GBDT declined considerably (Figure 3C-D, Table 2), yielding AUCs of 0.88 (95% CI: 0.78-0.98), 0.87 (95% CI: 0.75-0.98), and 0.89 (95% CI: 0.79-0.99), respectively. In contrast, LightGBM outperformed the other models, achieving an AUC of 0.90 (95% CI: 0.81-0.99) with highest PR-AUC of 0.70 (95% CI: 0.65-0.76). Its corresponding accuracy, sensitivity, specificity, PPV, and NPV were 0.88 (95% CI: 0.87-0.89), 0.57 (95% CI: 0.47-0.68), 0.93 (95% CI: 0.91-0.96), 0.62 (95% CI: 0.54-0.70), and 0.93 (95% CI: 0.91-0.94), respectively. Based on this comprehensive evaluation, LightGBM was selected as the final algorithm for model construction.<br>The LightGBM model was retrained on the training and validation cohorts, assessed on the test cohort. The LightGBM model achieved an AUC of 0.97 (95% CI: 0.96-0.98) on the training cohort and 0.89 (95% CI: 0.83-0.95) on the validation cohort. In the test cohort, the AUC remained consistent at 0.88 (Figure 3A-C).<br>The learning curve (Figure 4A) demonstrates that as the sample size increases, the model's predictive performance on the validation set improves progressively. This trend suggests that the LightGBM model exhibits good generalization ability and avoids both underfitting and overfitting. Moreover, it underscores the importance of large sample sizes in achieving stable predictions. The calibration curve (Figure 4B) indicates that the predicted probabilities closely align with the observed outcomes, with an expected calibration error of 0.064 (95% CI: 0.035-0.102). The DCA (Figure 4C) further illustrates that the model yields positive net clinical benefit across a wide range of threshold

				probabilities (0%–85%).
		(*b*) Report category boundaries when continuous variables were categorized	-	Not applicable
		(*c*) If relevant, consider translating estimates of relative risk into absolute risk for a meaningful time period		A total of 7,464 patients with upper urinary tract stones who underwent surgery were included in this study. Patients were divided into the experimental group (urosepsis, n = 622) and the control group (non-urosepsis, n = 6,842).
Other analyses	17	Report other analyses done—eg analyses of subgroups and interactions, and sensitivity analyses	13-14	The learning curve (Figure 4A) demonstrates that as the sample size increases, the model's predictive performance on the validation set improves progressively. This trend suggests that the LightGBM model exhibits good generalization ability and avoids both underfitting and overfitting. Moreover, it underscores the importance of large sample sizes in achieving stable predictions. The calibration curve (Figure 4B) indicates that the predicted probabilities closely align with the observed outcomes, with an expected calibration error of 0.064 (95% CI: 0.035-0.102). The DCA (Figure 4C) further illustrates that the model yields positive net clinical benefit across a wide range of threshold probabilities (0%–85%).<br>To enhance the interpretability of the model and facilitate clinical application by avoiding the "black box" effect, we employed SHAP analysis to visualize the contribution of each variable to the model's predictions. The results indicated that postoperative PCT/ALB, neutrophil, IL-6, ALB, and PT were the top five predictors of urosepsis (Figure 5A-B). Higher levels of postoperative PCT/ALB, neutrophil, IL-6, ALB, and PT were associated with an increased likelihood of urosepsis. We further evaluated the predictive performance of individual variables, which yielded results consistent with the SHAP analysis. Postoperative PCT/ALB, IL-6, and the HCT ratio emerged as the most predictive variables, with AUCs of 0.85, 0.78, and 0.73, respectively (Supplementary Figure 2). Additionally, postoperative PT and ALB each achieved AUCs of 0.70.
**Discussion**				
Key results	18	Summarise key results with reference to study objectives	16	In this study, we developed a predictive tool for urosepsis using multiple ML algorithms and comprehensive patient data collected from our hospital. We retrospectively analyzed data from 7,464 patients who underwent PCNL, RIRS, or URL. Following careful feature selection, eight clinical variables were ultimately included in model development. Eight ML algorithms were applied for model construction and validation. Among them, the

				LightGBM model demonstrated the highest predictive performance, with AUCs of 1.0, 0.90, and 0.88 in the training, validation, and test cohorts, respectively. The learning curve, calibration plot, and decision curve analysis indicated a good model fit, strong calibration, and substantial clinical benefit. Postoperative levels of PCT/ALB, neutrophil, IL-6, ALB, and PT were identified as key predictors using the SHAP algorithm. Furthermore, to evaluate the predictive power of individual variables, the AUCs of postoperative PCT/ALB, IL-6, and the HCT were 0.85, 0.78, and 0.73, respectively. An online platform based on the LightGBM model was also developed, enabling clinicians and patients to assess urosepsis risk and support timely intervention.
Limitations	19	Discuss limitations of the study, taking into account sources of potential bias or imprecision. Discuss both direction and magnitude of any potential bias	19-20	Despite its promising findings, this study has several limitations. First, as a retrospective analysis, it is subject to selection bias; thus, external validation using prospective, multi-center datasets is necessary to improve the generalizability of our results. Second, although the model incorporates essential laboratory variables, it lacks advanced biomolecular markers, which could enhance predictive accuracy. Imaging also plays a vital role in the early diagnosis and risk stratification of urosepsis by revealing anatomical abnormalities and complications [40]. Future studies should integrate radiomics and clinical data to improve prediction accuracy. Third, based on the strong performance of the LightGBM model, we developed an online platform for clinical use. However, variations in clinical feature measurements across institutions may affect the model's predictive performance. Efforts should focus on standardizing clinical parameters based on reference ranges. Fourth, while LightGBM performed well, the use of deep learning models incorporating longitudinal patient data may further improve risk predictions. An additional limitation pertains to the interpretability of the model. Although the SHAP algorithm provided transparent insights into the contribution of each predictor, we did not validate the robustness of SHAP values. This means the reported variable importance rankings may be susceptible to sample-specific randomness, and their generalizability to other datasets cannot be fully confirmed without further validation. Future research should address this by incorporating stability testing of SHAP attributions. Notably, the final model relies exclusively on postoperative parameters, allowing for timely risk stratification. However, this also limits the model's utility for preoperative risk assessment. Future investigations should explore integrating preoperative variables (e.g., preoperative urine culture results, anatomical factors) to develop a dual-stage prediction

				model for more comprehensive risk management. The last but not the least, although we included demographic and baseline clinical characteristics in the initial feature set, these variables were not retained in the final model due to non-significant group differences. However, adjusting for these factors may help control potential confounding and improve generalizability. Future studies could incorporate these variables as covariates in the model to validate their impact on predictive performance.
Interpretation	20	Give a cautious overall interpretation of results considering objectives, limitations, multiplicity of analyses, results from similar studies, and other relevant evidence	16-18	We carefully selected several parameters for model construction, including postoperative IL-6, SAA, PCT/ALB, NLPR, PT, ALB, HCT, and neutrophil. SAA is an acute-phase protein that increases sharply in plasma during inflammatory responses, such as those seen in sepsis. Its dynamic fluctuations are associated with patient prognosis, and combined measurement with other biomarkers such as CRP and PCT facilitates early identification of sepsis [23]. Consistent with previous studies, PCT remains a key biomarker for early diagnosis. Evidence suggests that elevated PCT levels can be detected early in patients with sepsis [24]. However, the diagnostic performance of PCT varies across studies, with reported AUC values ranging from 0.7 to 0.9. These discrepancies may be attributed to differences in patient cohorts and potential biases inherent in observational studies [25,26]. Notably, studies specifically focused on the diagnostic utility of PCT in urosepsis remain limited. In our study, PCT demonstrated an AUC of 0.81 for predicting urosepsis and was identified as the most important predictor by SHAP analysis. IL-6 is rapidly induced during the acute phase of infection or tissue injury and contributes to host defense by stimulating the production of acute-phase proteins and enhancing immune responses [27]. Combined detection of IL-6 and IL-10 has shown superior diagnostic accuracy for bacteremia and severe infections compared to PCT, particularly in pediatric patients with hematologic malignancies [28]. Zhang et al. reported that IL-6 achieved an AUC of 0.679 in differentiating Gram-positive from Gram-negative sepsis [29]. In a rat model of urosepsis, Cao et al. found that elevated IL-6 levels were significantly associated with multiple organ dysfunction [30].<br>Patients with sepsis commonly exhibit elevated WBC, neutrophils, and NLPR, along with lymphopenia, all of which are correlated with the severity of infection [31]. Neutrophil dysfunction, characterized by diminished elastase release and reduced reactive oxygen species production, is a hallmark of sepsis. Among neutrophil surface markers, CD64 has emerged as a highly promising biomarker, demonstrating significant elevation in sepsis

				and an AUC exceeding 0.9 in several studies [32,33]. HCT plays a critical role in determining blood viscosity and may contribute to sepsis-related organ dysfunction through microvascular obstruction. Although dynamic changes in HCT levels have been observed in patients with sepsis, its diagnostic value remains under investigation [34]. Cao et al. reported that serum albumin (ALB) levels were significantly lower in the sepsis group compared to the non-sepsis group, with an AUC of approximately 0.73 for sepsis diagnosis, consistent with our findings [35]. Sepsis is frequently associated with coagulation dysfunction, and prolonged PT is one of its characteristic manifestations. Prolonged PT indicates the consumption of coagulation factors in patients with sepsis, which results from excessive activation [36].<br>Interestingly, our differential analysis revealed that multiple preoperative laboratory parameters had already exhibited abnormalities. A high preoperative Systemic Immune-Inflammation Index (SII) has been identified as an independent risk factor for postoperative sepsis in patients undergoing surgery for intestinal obstruction [37]. Nedbal et al. also reported a positive association between postoperative sepsis and preoperative urine culture results [38]. However, Sui et al. argued that abnormal preoperative laboratory tests are not significant independent predictors of postoperative complications in low-risk ambulatory urological procedures, although this study did not include stone-related surgeries [39]. These findings suggest that patients who develop urosepsis may already exhibit preoperative pathophysiological changes, highlighting the potential for early prediction of urosepsis before surgery.
Generalisability	21	Discuss the generalisability (external validity) of the study results	19	First, as a retrospective analysis, it is subject to selection bias; thus, external validation using prospective, multi-center datasets is necessary to improve the generalizability of our results.
**Other information**				
Funding	22	Give the source of funding and the role of the funders for the present study and, if applicable, for the original study on which the present article is based	23	This work was supported by Guangxi Science and Technology Major Project (Grant No. GuikeAA22096032), Guangxi Science and Technology Major Project (Grant No. GuikeAA22096030) and Science Foundation for Distinguished Young Scholars of Guangxi (Grant No. 2023GXNSFFA026003).

*Give information separately for exposed and unexposed groups.

Note: An Explanation and Elaboration article discusses each checklist item and gives methodological background and published examples of transparent reporting. The STROBE checklist is best used in conjunction with this article (freely available on the Web sites of PLoS Medicine at http://www.plosmedicine.org/, Annals of Internal Medicine at http://www.annals.org/, and Epidemiology at http://www.epidem.com/). Information on the STROBE Initiative is available at http://www.strobe-statement.org.
