# Supplementary material for: Leveraging laboratory biomarkers to predict urosepsis after upper urinary tract stone surgery: an explainable machine learning approach
Source: BMC Med Inform Decis Mak. 2025 Dec 20;26:27. doi: 10.1186/s12911-025-03314-y (PMC12838489; doi:10.1186/s12911-025-03314-y)
Supplement: Supplementary file 4 — Supplementary Material 4 [file 12911_2025_3314_MOESM4_ESM.pdf]

Supplementary Table 2. Clinical Characteristics of Patients in the training cohort.

Variables	ALL (n = 7464)	Non-urosepsis (n = 6842)	Urosepsis (n = 622)	*P*-value
Age	54.00 (46.00. 63.00)	54.00 (46.00. 63.00)	56.00 (49.00. 64.00)	0.072
BMI	23.73 (21.47. 25.95)	23.78 (21.48. 25.97)	23.19 (20.83. 25.33)	<0.001
Times_operstion	1.00 (1.00. 2.00)	1.00 (1.00. 2.00)	1.00 (1.00. 2.00)	0.001
ASA				0.007
1	717 (15.13%)	676 (15.58%)	41 (10.25%)	
2	3918 (82.69%)	3571 (82.32%)	347 (86.75%)	
3	100 (2.11%)	89 (2.05%)	11 (2.75%)	
4	3 (0.06%)	2 (0.05%)	1 (0.25%)	
Barthel_Index	40.00 (30.00. 85.00)	40.00 (30.00. 85.00)	35.00 (25.00. 80.00)	0.03
VTE	2.00 (1.00. 3.00)	2.00 (1.00. 3.00)	2.00 (1.00. 4.00)	0.014
Morse	4.00 (4.00. 5.00)	4.00 (4.00. 5.00)	4.00 (4.00. 5.00)	0.989
Sex				<0.001
Female	1912 (40.03%)	1671 (38.19%)	241 (60.10%)	
Male	2864 (59.97%)	2704 (61.81%)	160 (39.90%)	
Diabetes				0.3
No	4418 (92.50%)	4053 (92.64%)	365 (91.02%)	
Yes	347 (7.27%)	311 (7.11%)	36 (8.98%)	
Hypertension				0.831
No	3760 (78.73%)	3442 (78.67%)	318 (79.30%)	
Yes	1004 (21.02%)	921 (21.05%)	83 (20.70%)	
U_culture				0.301
No	4128 (86.43%)	3783 (86.47%)	345 (86.03%)	
Yes	58 (1.21%)	50 (1.14%)	8 (2.00%)	
TRUE	590 (12.35%)	542 (12.39%)	48 (11.97%)	
U_NIT				<0.001
0	4546 (95.18%)	4194 (95.86%)	352 (87.78%)	
+	94 (1.97%)	74 (1.69%)	20 (4.99%)	

++	136 (2.85%)	107 (2.45%)	29 (7.23%)	
Type of operation				<0.001
PCNL	1871 (39.18%)	1640 (37.49%)	231 (57.61%)	
RIRS	1252 (26.21%)	1171 (26.77%)	81 (20.20%)	
URL	1653 (34.61%)	1564 (35.75%)	89 (22.19%)	
SG	1.01 (1.00. 1.01)	1.01 (1.00. 1.01)	1.01 (1.00. 1.01)	<0.001
PH_value	6.00 (6.00. 6.50)	6.00 (6.00. 6.50)	6.50 (6.00. 7.00)	0.007
U_LEU	1.00 (0.00. 3.00)	1.00 (0.00. 3.00)	2.00 (1.00. 4.00)	<0.001
U_PRO	0.00 (0.00. 1.00)	0.00 (0.00. 1.00)	0.00 (0.00. 1.00)	0.007
U_GLU	0.00 (0.00. 0.00)	0.00 (0.00. 0.00)	0.00 (0.00. 0.00)	0.739
U_URO				0.583
0	4740 (99.25%)	4342 (99.25%)	398 (99.25%)	
+	21 (0.44%)	19 (0.43%)	2 (0.50%)	
++	9 (0.19%)	9 (0.21%)	0 (0.00%)	
+++	6 (0.13%)	5 (0.11%)	1 (0.25%)	
U_BIL				0.074
0	4744 (99.33%)	4349 (99.41%)	395 (98.50%)	
+	16 (0.34%)	12 (0.27%)	4 (1.00%)	
++	8 (0.17%)	7 (0.16%)	1 (0.25%)	
+++	8 (0.17%)	7 (0.16%)	1 (0.25%)	
OB	2.00 (0.00. 3.00)	2.00 (0.00. 3.00)	2.00 (0.00. 3.00)	0.856
SED_RBC	58.50 (12.00. 552.00)	59.00 (12.00. 566.00)	48.00 (12.00. 430.00)	0.375
SED_WBC	46.00 (14.00. 173.00)	41.50 (13.05. 153.00)	133.00 (33.10. 532.00)	<0.001
SED_EC	5.00 (2.00. 11.00)	5.00 (2.00. 11.00)	5.00 (2.00. 12.00)	0.625
SED_casts	0.00 (0.00. 1.00)	0.00 (0.00. 1.00)	0.00 (0.00. 1.00)	0.149
SED_bacteria	25.00 (10.00. 95.00)	24.00 (9.00. 86.00)	45.00 (15.00. 246.00)	<0.001
U_conductivity	12.20 (8.70. 15.90)	12.40 (8.90. 16.10)	10.50 (7.80. 13.80)	<0.001
WBC	6.94 (5.83. 8.34)	6.91 (5.81. 8.30)	7.25 (6.08. 8.72)	0.008
Neut%	60.40 (54.50. 66.40)	60.30 (54.42. 66.30)	60.70 (54.70. 67.30)	0.201

Lymph%	27.90 (22.40. 33.10)	27.90 (22.50. 33.10)	27.70 (21.50. 33.30)	0.308
Mono%	6.90 (5.80. 8.20)	6.90 (5.90. 8.20)	6.70 (5.60. 8.40)	0.288
Eos%	3.10 (1.90. 4.70)	3.10 (1.90. 4.70)	3.10 (1.90. 4.80)	0.95
Baso%	0.60 (0.40. 0.80)	0.60 (0.40. 0.80)	0.60 (0.40. 0.80)	0.077
Neut	4.12 (3.29. 5.26)	4.10 (3.28. 5.22)	4.35 (3.38. 5.47)	0.014
Lymph	1.87 (1.49. 2.27)	1.86 (1.49. 2.27)	1.91 (1.50. 2.35)	0.155
Mono	0.48 (0.38. 0.60)	0.48 (0.38. 0.59)	0.49 (0.38. 0.63)	0.325
Eos	0.21 (0.13. 0.33)	0.21 (0.13. 0.33)	0.22 (0.13. 0.34)	0.303
Baso	0.04 (0.03. 0.06)	0.04 (0.03. 0.06)	0.04 (0.03. 0.06)	0.728
NRBC	0.00 (0.00. 0.00)	0.00 (0.00. 0.00)	0.00 (0.00. 0.00)	0.46
NRBC/RBC	0.00 (0.00. 0.00)	0.00 (0.00. 0.00)	0.00 (0.00. 0.00)	0.487
RBC	4.56 (4.15. 5.00)	4.58 (4.17. 5.02)	4.36 (3.98. 4.77)	<0.001
HGB	130.00 (117.00. 142.00)	130.00 (117.00. 143.00)	123.00 (108.00. 135.00)	<0.001
HCT	0.39 (0.36. 0.43)	0.40 (0.36. 0.43)	0.37 (0.34. 0.41)	<0.001
MCV	87.80 (83.50. 91.00)	87.80 (83.60. 91.00)	86.80 (82.90. 90.20)	0.013
MCH	29.20 (27.30. 30.50)	29.30 (27.40. 30.50)	28.70 (26.80. 30.10)	<0.001
MCHC	331.00 (320.00. 339.00)	331.00 (321.00. 339.00)	329.00 (318.00. 337.00)	0.001
RDW	12.70 (12.10. 13.70)	12.70 (12.00. 13.60)	13.00 (12.20. 14.20)	<0.001
PLT	266.00 (224.00. 315.00)	264.00 (223.00. 312.00)	286.00 (239.00. 342.00)	<0.001
MPV	9.70 (9.20. 10.20)	9.70 (9.20. 10.20)	9.60 (9.10. 10.10)	0.045
Plateletcrit	0.26 (0.22. 0.30)	0.26 (0.22. 0.30)	0.28 (0.24. 0.32)	<0.001
BUA	365.00 (303.00. 438.52)	366.00 (303.25. 439.00)	360.10 (300.90. 431.70)	0.442
BUN	5.30 (4.30. 6.61)	5.29 (4.30. 6.59)	5.41 (4.20. 7.06)	0.3
Scr	96.00 (79.20. 122.40)	96.00 (79.67. 122.00)	96.00 (78.00. 136.00)	0.536
eGFR	73.92 (55.93. 89.33)	74.27 (56.45. 89.56)	70.23 (50.47. 87.83)	0.004
HCO3	23.80 (22.10. 25.40)	23.80 (22.10. 25.40)	23.40 (21.30. 25.00)	0.001
β2_MG	2.27 (1.88. 2.95)	2.25 (1.86. 2.90)	2.56 (2.06. 3.76)	<0.001
CysC	1.12 (0.99. 1.37)	1.12 (0.98. 1.36)	1.20 (1.02. 1.59)	<0.001
TP	70.48 (67.30. 73.80)	70.30 (67.20. 73.70)	72.00 (68.50. 75.50)	<0.001

ALB	40.00 (37.90. 42.10)	40.10 (37.90. 42.10)	39.50 (36.60. 41.50)	<0.001
GLB	30.20 (27.60. 33.50)	30.08 (27.50. 33.20)	32.60 (29.42. 36.30)	<0.001
ALB/GLB	1.30 (1.20. 1.50)	1.30 (1.20. 1.50)	1.20 (1.10. 1.40)	<0.001
BIL	7.50 (5.40. 10.30)	7.50 (5.50. 10.30)	6.70 (4.80. 9.40)	<0.001
DBIL	3.50 (2.60. 4.50)	3.50 (2.64. 4.60)	3.20 (2.40. 4.30)	<0.001
IBil	4.00 (2.70. 5.70)	4.00 (2.70. 5.80)	3.50 (2.20. 5.00)	<0.001
GPT	16.00 (11.10. 23.00)	16.00 (11.30. 23.00)	15.00 (10.60. 22.00)	0.061
GOT	18.00 (15.00. 22.00)	18.00 (15.00. 22.00)	18.00 (14.40. 22.90)	0.368
ALP	72.00 (61.00. 86.00)	71.00 (60.00. 85.00)	76.00 (63.00. 91.00)	<0.001
GGT	25.67 (17.08. 40.00)	25.20 (17.00. 40.00)	27.00 (18.00. 40.00)	0.244
TBA	4.50 (2.80. 7.50)	4.50 (2.80. 7.51)	4.40 (2.70. 7.30)	0.328
CRP	2.51 (1.11. 7.02)	2.44 (1.07. 6.71)	3.70 (1.62. 10.72)	<0.001
K	3.89 (3.64. 4.13)	3.89 (3.64. 4.13)	3.87 (3.60. 4.11)	0.164
Ca	2.28 (2.22. 2.34)	2.28 (2.22. 2.34)	2.29 (2.23. 2.36)	0.046
PT	10.90 (10.50. 11.50)	10.90 (10.40. 11.50)	11.00 (10.50. 11.50)	0.043
INR	0.95 (0.90. 1.00)	0.95 (0.90. 1.00)	0.95 (0.91. 1.00)	0.087
PTA	114.00 (101.00. 127.00)	114.00 (101.00. 128.00)	113.20 (97.00. 126.00)	0.016
TT	18.62 (17.80. 19.50)	18.62 (17.80. 19.50)	18.60 (17.60. 19.40)	0.011
APTT	25.90 (23.50. 28.70)	25.90 (23.50. 28.70)	25.70 (23.40. 28.60)	0.519
PF	3.09 (2.61. 3.88)	3.06 (2.59. 3.82)	3.44 (2.92. 4.61)	<0.001
NLR	2.17 (1.65. 2.94)	2.17 (1.65. 2.92)	2.17 (1.64. 3.10)	0.254
PLR	141.39 (111.86. 182.49)	140.72 (111.76. 180.92)	151.08 (112.23. 204.97)	0.002
LMR	4.00 (3.02. 5.11)	4.00 (3.03. 5.09)	3.95 (2.90. 5.32)	0.848
ELR	0.11 (0.07. 0.17)	0.11 (0.07. 0.17)	0.11 (0.07. 0.18)	0.747
dNLR	1.52 (1.20. 1.98)	1.52 (1.19. 1.97)	1.55 (1.21. 2.06)	0.208
NLPR	0.01 (0.01. 0.01)	0.01 (0.01. 0.01)	0.01 (0.01. 0.01)	0.014
SII	577.34 (409.40. 831.31)	573.03 (406.96. 821.20)	633.64 (452.91. 986.28)	<0.001
AISI	273.61 (173.56. 444.33)	270.60 (172.66. 437.42)	299.43 (194.41. 531.11)	0.003
SIRI	1.01 (0.70. 1.58)	1.00 (0.69. 1.56)	1.09 (0.70. 1.74)	0.141

LCR	0.75 (0.26. 1.72)	0.78 (0.27. 1.77)	0.53 (0.17. 1.30)	<0.001
CRP/ALB	0.06 (0.03. 0.18)	0.06 (0.03. 0.17)	0.10 (0.04. 0.27)	<0.001
p_CRP	2.44 (0.85. 7.78)	2.16 (0.78. 6.78)	9.49 (2.50. 40.50)	<0.001
p_SAA	10.00 (7.00. 25.00)	10.00 (6.00. 20.00)	32.00 (10.50. 271.00)	<0.001
p_PCT	0.05 (0.03. 0.09)	0.05 (0.03. 0.08)	0.23 (0.08. 5.59)	<0.001
p_IL-6	14.03 (6.38. 36.84)	12.59 (5.95. 29.74)	88.36 (21.35. 543.40)	<0.001
p_WBC	8.14 (6.41. 10.30)	8.00 (6.37. 9.96)	12.14 (6.92. 14.73)	<0.001
p_Neut%	71.70 (62.40. 81.60)	70.90 (62.00. 80.40)	81.80 (69.00. 89.80)	<0.001
p_Lymph%	19.70 (12.50. 27.55)	20.30 (13.20. 28.13)	11.60 (6.10. 20.70)	<0.001
p_Mono%	5.60 (3.50. 7.10)	5.70 (3.70. 7.10)	4.90 (2.60. 7.00)	<0.001
p_Eos%	1.40 (0.60. 2.70)	1.50 (0.70. 2.70)	0.80 (0.20. 2.20)	<0.001
p_Baso%	0.40 (0.30. 0.60)	0.40 (0.30. 0.60)	0.30 (0.20. 0.40)	<0.001
p_Neut	5.66 (4.16. 7.89)	5.55 (4.12. 7.57)	9.39 (5.00. 12.79)	<0.001
p_Lymph	1.50 (1.02. 2.11)	1.54 (1.05. 2.13)	1.15 (0.72. 1.73)	<0.001
p_Mono	0.43 (0.27. 0.60)	0.43 (0.27. 0.59)	0.48 (0.24. 0.77)	<0.001
p_Eos	0.11 (0.05. 0.21)	0.11 (0.05. 0.21)	0.08 (0.02. 0.19)	<0.001
p_Baso	0.03 (0.02. 0.04)	0.03 (0.02. 0.04)	0.03 (0.02. 0.05)	0.038
p_RBC	4.36 (3.89. 4.83)	4.39 (3.94. 4.85)	3.92 (3.44. 4.42)	<0.001
p_HGB	123.00 (110.00. 137.00)	125.00 (112.00. 137.00)	110.00 (95.00. 125.00)	<0.001
p_HCT	0.37 (0.34. 0.41)	0.38 (0.34. 0.41)	0.34 (0.29. 0.38)	<0.001
p_MCV	87.70 (83.30. 91.20)	87.80 (83.50. 91.30)	86.50 (81.70. 90.35)	0.001
p_MCH	29.30 (27.40. 30.60)	29.40 (27.48. 30.60)	28.90 (26.90. 30.10)	<0.001
p_MCHC	331.00 (320.00. 340.00)	331.00 (320.00. 340.00)	330.00 (320.00. 340.00)	0.629
p_RDW	12.70 (12.00. 13.70)	12.60 (12.00. 13.60)	13.10 (12.40. 14.40)	<0.001
p_PLT	250.00 (208.00. 301.00)	251.00 (209.00. 301.00)	245.00 (192.00. 304.50)	0.114
p_MPV	9.70 (9.20. 10.20)	9.70 (9.20. 10.20)	9.70 (9.20. 10.30)	0.319
p_PCT	0.24 (0.20. 0.29)	0.24 (0.20. 0.29)	0.24 (0.20. 0.30)	0.492
p_BUA	264.10 (197.90. 338.95)	265.10 (199.00. 339.50)	253.90 (183.07. 326.90)	0.019
p_BUN	4.20 (3.30. 5.40)	4.20 (3.30. 5.30)	4.30 (3.20. 6.00)	0.084

p_Scr	94.00 (76.00. 118.00)	93.00 (76.00. 117.00)	102.00 (76.00. 140.50)	<0.001
p_eGFR	75.44 (55.38. 94.77)	76.42 (56.65. 94.99)	61.99 (40.92. 88.23)	<0.001
p_HCO3	23.10 (21.20. 24.90)	23.20 (21.30. 25.00)	22.00 (20.30. 23.90)	<0.001
p_β2-MG	2.13 (1.68. 2.84)	2.09 (1.66. 2.76)	2.71 (2.06. 4.14)	<0.001
p_TP	65.50 (61.50. 69.20)	65.60 (61.60. 69.20)	63.00 (58.45. 68.97)	<0.001
p_ALB	36.30 (33.90. 38.70)	36.50 (34.12. 38.80)	33.60 (30.60. 36.27)	<0.001
p_GLB	28.80 (26.10. 31.80)	28.70 (26.10. 31.70)	29.60 (25.92. 33.00)	0.013
p_ALB/GLB	1.30 (1.10. 1.40)	1.30 (1.10. 1.40)	1.10 (1.00. 1.30)	<0.001
p_BIL	10.10 (7.30. 13.60)	10.20 (7.40. 13.70)	9.65 (6.90. 13.00)	0.115
p_DBIL	4.60 (3.50. 6.00)	4.60 (3.50. 6.00)	4.90 (3.73. 6.70)	0.001
p_IBil	5.40 (3.60. 7.60)	5.50 (3.70. 7.80)	4.50 (2.90. 6.50)	<0.001
p_GPT	14.00 (10.00. 21.00)	14.00 (10.00. 20.70)	15.00 (10.00. 22.00)	0.067
p_GOT	16.25 (14.00. 20.20)	16.00 (13.70. 20.00)	19.00 (15.00. 28.40)	<0.001
p_ALP	65.00 (54.00. 77.00)	65.00 (54.00. 77.00)	63.00 (51.00. 79.00)	0.168
p_GGT	25.00 (16.90. 40.40)	24.00 (16.00. 39.00)	33.00 (20.00. 54.00)	<0.001
p_TBA	1.80 (1.00. 3.40)	1.80 (1.00. 3.30)	2.20 (1.20. 4.10)	<0.001
p_K	3.84 (3.57. 4.11)	3.85 (3.59. 4.13)	3.67 (3.40. 3.96)	<0.001
p_Ca	2.18 (2.10. 2.26)	2.18 (2.10. 2.26)	2.14 (2.04. 2.23)	<0.001
p_PT	11.70 (11.20. 12.30)	11.70 (11.10. 12.20)	12.20 (11.60. 13.40)	<0.001
p_INR	1.02 (0.97. 1.07)	1.02 (0.96. 1.06)	1.06 (1.01. 1.17)	<0.001
p_PTA	103.00 (91.00. 114.00)	103.00 (93.00. 116.00)	93.00 (74.00. 106.00)	<0.001
p_TT	18.50 (17.58. 19.50)	18.50 (17.60. 19.50)	18.10 (17.10. 19.40)	0.006
p_APTT	26.40 (23.50. 29.50)	26.40 (23.50. 29.15)	27.30 (23.10. 33.10)	0.012
p_PF	3.11 (2.53. 3.93)	3.08 (2.53. 3.86)	3.50 (2.70. 4.47)	0.004
p_NLR	3.64 (2.28. 6.52)	3.50 (2.22. 6.09)	6.88 (3.34. 14.64)	<0.001
p_PLR	165.97 (116.67. 247.78)	162.50 (115.11. 241.21)	214.68 (141.57. 328.46)	<0.001
p_LMR	3.97 (2.60. 6.03)	4.09 (2.73. 6.11)	2.65 (1.47. 4.53)	<0.001
p_ELR	0.07 (0.04. 0.13)	0.07 (0.04. 0.13)	0.07 (0.03. 0.15)	0.503
p_dNLR	2.53 (1.66. 4.44)	2.44 (1.63. 4.10)	4.51 (2.22. 8.81)	<0.001

p_NLPR	0.01 (0.01. 0.03)	0.01 (0.01. 0.02)	0.03 (0.01. 0.07)	<0.001
p_SII	925.00 (551.56. 1674.08)	890.39 (538.36. 1556.36)	1736.00 (872.95. 3241.35)	<0.001
p_AISI	336.41 (188.84. 668.96)	320.28 (185.51. 609.26)	796.48 (294.00. 1680.15)	<0.001
p_LCR	0.59 (0.16. 1.87)	0.67 (0.19. 2.01)	0.10 (0.02. 0.49)	<0.001
p_SIRI	1.32 (0.77. 2.47)	1.26 (0.76. 2.28)	3.04 (1.18. 7.06)	<0.001
p_CRP/ALB	0.07 (0.02. 0.23)	0.06 (0.02. 0.19)	0.31 (0.08. 1.22)	<0.001
p_PCT/ALB	<0.01 (<0.01. <0.01)	<0.01 (<0.01. <0.01)	0.01 (<0.01. 0.13)	<0.001
WBC-p_WBC	0.86 (0.68. 1.08)	0.87 (0.70. 1.09)	0.66 (0.50. 0.97)	<0.001

U_LEU, urine leukocytes. U_NIT, urine nitrite. U_PRO, urine protein. SED_WBC, sediment white blood cells. SED_bacteria, sediment bacteria. WBC, white blood count. Neut%, neutrophil percentage. Neut, absolute neutrophil count. Mono, monocyte count. RDW, red cell distribution width. β2 MG, β2 microglobulin. CysC, cystatin C. TP, total protein. GLB, globulin. ALP, alkaline phosphatase. GGT, gamma-glutamyl transferase. SAA, Serum amyloid A. PCT, Procalcitonin. IL-6, Interleukin-6. CRP, C-reactive protein. PT, prothrombin time. INR, international normalized ratio. PF, partial thromboplastin time. SED_EC, sediment erythrocytes. Lymph%, lymphocyte percentage. HGB, hemoglobin. HCT, hematocrit. MCH, mean corpuscular hemoglobin. eGFR, estimated glomerular filtration rate. ALB, albumin. IBil, indirect bilirubin. NLR, neutrophil-to-lymphocyte ratio. dNLR, derived NLR. PLR, platelet-to-lymphocyte ratio. LMR, lymphocyte-to-monocyte ratio. ELR, eosinophil-to-lymphocyte ratio. NLPR, neutrophil-lymphocyte-platelet ratio. SII, systemic immune-inflammation index. AISI, aggregate index of systemic inflammation. SIRI, systemic inflammation response index. LCR, lymphocyte-to-CRP ratio. CRP/ALB, CRP-to-albumin ratio. p_, postoperative.
