# Supplementary material for: Leveraging laboratory biomarkers to predict urosepsis after upper urinary tract stone surgery: an explainable machine learning approach
Source: BMC Med Inform Decis Mak. 2025 Dec 20;26:27. doi: 10.1186/s12911-025-03314-y (PMC12838489; doi:10.1186/s12911-025-03314-y)
Supplement: Supplementary file 5 — Supplementary Material 5 [file 12911_2025_3314_MOESM5_ESM.pdf]

Supplementary Table 3. Result of LASSO Regression.

Variables	Mean squared error	Standard error coefficient of minimum distance
(Intercept)	7.747	-0.454
Type of operation	0	0
U_NIT	0.015	0
Sex	0	0
BMI	0	0
Times_operstion	0	0
SG	-9.667	0
PH_value	0	0
U_LEU	0.011	0
U_PRO	0	0
SED_WBC	0	0
SED_bacteria	0	0
U_conductivity	0	0
WBC	0	0
Neut	0	0
RBC	0	0
HGB	0	0
HCT	0	0
MCV	0	0
MCH	0	0
MCHC	0	0
RDW	0	0
PLT	0	0
MPV	0	0
Plateletcrit	0	0
eGFR	0	0
HCO3	0	0

β2_MG	0	0
CysC	0	0
TP	0	0
ALB	0	0
GLB	0	0
ALB/GLB	0	0
BIL	0	0
DBIL	0	0
I_Bil	0	0
ALP	0	0
CRP	0	0
Ca	0	0
PT	0	0
PTA	0	0
TT	0	0
PF	0	0
PLR	0	0
SII	0	0
AISI	0	0
LCR	0	0
CRP/ALB	0	0
p_CRP	0	0
p_SAA	0.001	0.001
p_PCT	0	0
p_IL6	0.001	0
p_WBC	0.004	0
p_Neut%	0	0
p_Lymph%	0	0
p_Mono%	0	0

p_Eos%	0	0
p_Baso%	0	0
p_Neut	0.131	0.072
p_Lymph	0	0
p_Mono	0	0
p_Eos	0	0
p_Baso	0	0
p_RBC	0	0
p_HGB	0	0
p_HCT	-6.654	-4.664
p_MCV	0	0
p_MCH	0	0
p_RDW	0	0
p_BUA	0	0
p_Scr	0	0
p_eGFR	0	0
p_HCO3	0	0
p_β2_MG	0.084	0
p_TP	-0.002	0
p_ALB	-0.02	-0.014
p_GLB	0	0
p_ALB/GLB	0	0
p_DBIL	0.039	0
p_IBil	0	0
p_GOT	0.001	0
p_GGT	0	0
p_TBA	0	0
p_K	0	0
p_Ca	0	0

p_PT	0.132	0.002
p_INR	0	0
p_PTA	0	0
p_TT	0	0
p_APTT	0	0
p_PF	0	0
p_NLR	0	0
p_PLR	0	0
p_LMR	0	0
p_dNLR	0	0
p_NLPR	0	0.31
p_SII	0	0
p_AISI	0	0
p_LCR	0	0
p_SIRI	0	0
p_CRP/ALB	0	0
p_PCT/ALB	0.475	0.409
WBC/WBC	0	0
ASA	0	0
