# Supplementary material for: Leveraging laboratory biomarkers to predict urosepsis after upper urinary tract stone surgery: an explainable machine learning approach
Source: BMC Med Inform Decis Mak. 2025 Dec 20;26:27. doi: 10.1186/s12911-025-03314-y (PMC12838489; doi:10.1186/s12911-025-03314-y)
Supplement: Supplementary file 6 — Supplementary Material 6 [file 12911_2025_3314_MOESM6_ESM.pdf]

Supplementary Table 4. Pearson Correlation Coefficient between Variables.

Variables	Post-SAA	Post-IL-6	Post-Neut	Post-HCT	Post-ALB	Post-PT	Post-NLPR	Post-PCT/ALB
**Post-SAA**	1	-0.01	0.19	-0.24	-0.25	0.15	0.07	0.25
**Post-IL-6**	-0.01	1	0.13	-0.17	-0.27	0.26	0.36	0.28
**Post-Neut**	0.19	0.13	1	0.02	-0.01	-0.02	0.61	0.32
**Post-HCT**	-0.24	-0.17	0.02	1	0.5	-0.3	-0.15	-0.22
**Post-ALB**	-0.25	-0.27	-0.01	0.5	1	-0.36	-0.21	-0.22
**Post-PT**	0.15	0.26	-0.02	-0.3	-0.36	1	0.2	0.15
**Post-NLPR**	0.07	0.36	0.61	-0.15	-0.21	0.2	1	0.32
**Post-PCT/ALB**	0.25	0.28	0.32	-0.22	-0.22	0.15	0.32	1
