# Supplementary material for: Leveraging laboratory biomarkers to predict urosepsis after upper urinary tract stone surgery: an explainable machine learning approach
Source: BMC Med Inform Decis Mak. 2025 Dec 20;26:27. doi: 10.1186/s12911-025-03314-y (PMC12838489; doi:10.1186/s12911-025-03314-y)
Supplement: Supplementary file 7 — Supplementary Material 7 [file 12911_2025_3314_MOESM7_ESM.pdf]

Supplementary Table 5. *P* Value of Pearson Correlation Analysis.

Variables	Post-SAA	Post-IL-6	Post-Neut	Post-HCT	Post-ALB	Post-PT	Post-NLPR	Post-PCT/ALB
**Post-SAA**	0	0.77	0	0	0	0	0.06	0
**Post-IL-6**	0.77	0	0	0	0	0	0	0
**Post-Neut**	0	0	0	0.53	0.71	0.58	0	0
**Post-HCT**	0	0	0.53	0	0	0	0	0
**Post-ALB**	0	0	0.71	0	0	0	0	0
**Post-PT**	0	0	0.58	0	0	0	0	0
**Post-NLPR**	0.06	0	0	0	0	0	0	0
**Post-PCT/ALB**	0	0	0	0	0	0	0	0
