# Supplementary material for: Leveraging laboratory biomarkers to predict urosepsis after upper urinary tract stone surgery: an explainable machine learning approach
Source: BMC Med Inform Decis Mak. 2025 Dec 20;26:27. doi: 10.1186/s12911-025-03314-y (PMC12838489; doi:10.1186/s12911-025-03314-y)
Supplement: Supplementary file 8 — Supplementary Material 8 [file 12911_2025_3314_MOESM8_ESM.pdf]

Supplementary Table 6. Clinical Characteristics of Patients in Training, Validation and test Cohorts.

Variables	Training cohort (n = 4776)	Validation cohort (n = 1195)	Test cohort (n = 1493)	P-value
Urosepsis				0.736
No	4375 (91.60%)	1102 (92.22%)	1365 (91.43%)	
Yes	401 (8.40%)	93 (7.78%)	128 (8.57%)	
Age	54.00 (46.00, 63.00)	54.00 (45.00, 62.00)	54.00 (46.00, 63.00)	0.479
BMI	23.73 (21.47, 25.95)	23.78 (21.59, 25.97)	23.78 (21.45, 25.86)	0.725
Times of operation	1.00 (1.00, 2.00)	1.00 (1.00, 2.00)	1.00 (1.00, 2.00)	0.649
Type of operation				0.045
PCNL	1871 (39.18%)	475 (39.75%)	650 (43.54%)	
RIRS	1252 (26.21%)	308 (25.77%)	350 (23.44%)	
URL	1653 (34.61%)	412 (34.48%)	493 (33.02%)	
Sex				0.69
Female	1912 (40.03%)	484 (40.50%)	582 (38.98%)	
Male	2864 (59.97%)	711 (59.50%)	911 (61.02%)	
Diabetes				0.252
No	4418 (92.50%)	1121 (93.81%)	1380 (92.43%)	
Yes	347 (7.27%)	72 (6.03%)	113 (7.57%)	
Hypertension				0.102
No	3760 (78.73%)	940 (78.66%)	1140 (76.36%)	
Yes	1004 (21.02%)	253 (21.17%)	353 (23.64%)	
ASA				0.669
1	717 (15.13%)	166 (13.95%)	220 (14.88%)	
2	3918 (82.69%)	999 (83.95%)	1227 (83.02%)	
3	100 (2.11%)	25 (2.10%)	31 (2.10%)	
4	3 (0.06%)	0 (0.00%)	0 (0.00%)	
Barthel_Index	40.00 (30.00, 85.00)	40.00 (30.00, 85.00)	40.00 (30.00, 85.00)	0.128
VTE	2.00 (1.00, 3.00)	2.00 (1.00, 3.00)	2.00 (1.00, 3.00)	0.015
Morse	4.00 (4.00, 5.00)	4.00 (4.00, 5.00)	4.00 (4.00, 5.00)	0.529

U_NIT, N (%):				0.4
0	4546 (95.18%)	1138 (95.23%)	1422 (95.24%)	
+	94 (1.97%)	28 (2.34%)	38 (2.55%)	
++	136 (2.85%)	29 (2.43%)	33 (2.21%)	
SG	1.01 (1.00, 1.01)	1.01 (1.00, 1.01)	1.01 (1.00, 1.01)	0.872
PH_value	6.00 (6.00, 6.50)	6.00 (6.00, 6.50)	6.00 (6.00, 6.50)	0.158
U_LEU	1.00 (0.00, 3.00)	1.00 (0.00, 3.00)	1.00 (0.00, 3.00)	0.677
U_PRO	0.00 (0.00, 1.00)	0.00 (0.00, 1.00)	0.00 (0.00, 1.00)	0.883
U_GLU	0.00 (0.00, 0.00)	0.00 (0.00, 0.00)	0.00 (0.00, 0.00)	0.981
U_URO	0.00 (0.00, 1.00)	0.00 (0.00, 1.00)	0.00 (0.00, 1.00)	0.153
U_BIL	0.00 (0.00, 1.00)	0.00 (0.00, 1.00)	0.00 (0.00, 1.00)	0.54
OB	2.00 (0.00, 3.00)	2.00 (0.00, 3.00)	2.00 (0.00, 3.00)	0.271
SED_RBC	58.50 (12.00, 552.00)	49.00 (11.00, 511.00)	72.00 (12.00, 554.00)	0.265
SED_WBC	46.00 (14.00, 173.00)	41.00 (12.00, 146.00)	48.00 (14.00, 170.00)	0.048
SED_EC	5.00 (2.00, 11.00)	5.00 (3.00, 11.00)	5.00 (2.00, 12.00)	0.324
SED_casts	0.00 (0.00, 1.00)	0.00 (0.00, 1.00)	0.00 (0.00, 1.00)	0.129
SED_bacteria	25.00 (10.00, 95.00)	21.00 (8.00, 70.00)	26.00 (9.00, 95.00)	0.002
U_conductivity	12.20 (8.70, 15.90)	12.10 (8.50, 15.40)	12.40 (8.90, 16.10)	0.162
WBC	6.94 (5.83, 8.34)	6.97 (5.82, 8.34)	7.05 (5.93, 8.44)	0.168
Neut%	60.40 (54.50, 66.40)	59.80 (53.70, 66.20)	60.40 (54.50, 66.50)	0.486
Lymph%	27.90 (22.40, 33.10)	28.50 (22.35, 34.00)	28.00 (22.44, 33.30)	0.278
Mono%	6.90 (5.80, 8.20)	6.90 (5.85, 8.20)	6.80 (5.80, 8.00)	0.259
Eos%	3.10 (1.90, 4.70)	3.00 (1.80, 4.60)	3.00 (1.90, 4.70)	0.279
Baso%	0.60 (0.40, 0.80)	0.60 (0.50, 0.80)	0.60 (0.40, 0.80)	0.122
Neut	4.12 (3.29, 5.26)	4.13 (3.26, 5.18)	4.21 (3.32, 5.27)	0.188
Lymph	1.87 (1.49, 2.27)	1.88 (1.51, 2.31)	1.88 (1.51, 2.34)	0.239
Mono	0.48 (0.38, 0.60)	0.48 (0.38, 0.61)	0.48 (0.38, 0.61)	0.757
Eos	0.21 (0.13, 0.33)	0.20 (0.12, 0.31)	0.21 (0.13, 0.33)	0.239
Baso	0.04 (0.03, 0.06)	0.04 (0.03, 0.06)	0.04 (0.03, 0.06)	0.435

RBC	4.56 (4.15, 5.00)	4.57 (4.15, 5.06)	4.56 (4.13, 5.03)	0.709
HGB	130.00 (117.00, 142.00)	129.00 (117.00, 143.00)	129.00 (117.00, 142.00)	0.864
HCT	0.39 (0.36, 0.43)	0.39 (0.36, 0.43)	0.39 (0.36, 0.43)	0.823
MCV	87.80 (83.50, 91.00)	87.40 (83.50, 90.80)	87.40 (83.20, 91.00)	0.266
MCH	29.20 (27.30, 30.50)	29.10 (27.30, 30.40)	29.20 (27.30, 30.50)	0.41
MCHC	331.00 (320.00, 339.00)	330.00 (320.50, 339.00)	330.60 (321.00, 339.00)	0.91
RDW	12.70 (12.10, 13.70)	12.70 (12.00, 13.60)	12.70 (12.10, 13.64)	0.623
PLT	266.00 (224.00, 315.00)	266.00 (227.50, 315.50)	266.00 (228.00, 314.00)	0.563
MPV	9.70 (9.20, 10.20)	9.60 (9.20, 10.20)	9.70 (9.20, 10.20)	0.301
Plateletcrit	0.26 (0.22, 0.30)	0.26 (0.22, 0.30)	0.26 (0.22, 0.30)	0.465
BUA	365.00 (303.00, 438.52)	367.40 (303.00, 439.20)	373.00 (305.00, 441.00)	0.182
BUN	5.30 (4.30, 6.61)	5.15 (4.17, 6.47)	5.38 (4.30, 6.80)	0.013
Scr	96.00 (79.20, 122.40)	95.00 (78.42, 121.00)	98.00 (79.00, 125.00)	0.31
eGFR	73.92 (55.93, 89.33)	75.01 (55.58, 89.41)	73.01 (54.16, 89.36)	0.241
HCO3	23.80 (22.10, 25.40)	23.90 (22.30, 25.30)	23.80 (22.20, 25.40)	0.781
β2_MG	2.27 (1.88, 2.95)	2.24 (1.83, 2.89)	2.30 (1.89, 2.99)	0.055
CysC	1.12 (0.99, 1.37)	1.13 (0.98, 1.38)	1.13 (0.99, 1.42)	0.292
TP	70.48 (67.30, 73.80)	70.40 (67.00, 73.95)	70.50 (67.30, 73.90)	0.684
ALB	40.00 (37.90, 42.10)	39.90 (37.70, 42.20)	40.10 (37.80, 42.20)	0.808
GLB	30.20 (27.60, 33.50)	30.20 (27.50, 33.50)	30.40 (27.70, 33.40)	0.609
ALB/GLB	1.30 (1.20, 1.50)	1.30 (1.20, 1.50)	1.30 (1.20, 1.50)	0.736
BIL	7.50 (5.40, 10.30)	7.70 (5.60, 10.70)	7.40 (5.40, 10.10)	0.069
DBIL	3.50 (2.60, 4.50)	3.50 (2.70, 4.75)	3.40 (2.60, 4.50)	0.054
IBil	4.00 (2.70, 5.70)	4.10 (2.80, 5.90)	4.00 (2.70, 5.60)	0.151
GPT	16.00 (11.10, 23.00)	16.00 (11.00, 22.70)	15.50 (11.26, 23.00)	0.862
GOT	18.00 (15.00, 22.00)	18.00 (15.00, 22.00)	18.00 (15.00, 22.00)	0.454
ALP	72.00 (61.00, 86.00)	70.00 (60.00, 85.00)	73.00 (61.00, 86.80)	0.085
GGT	25.67 (17.08, 40.00)	25.30 (18.00, 40.00)	25.00 (18.00, 38.00)	0.745
TBA	4.50 (2.80, 7.50)	4.70 (2.70, 7.60)	4.60 (2.70, 7.50)	0.586

CRP	2.51 (1.11, 7.02)	2.31 (1.10, 7.13)	2.64 (1.20, 7.19)	0.365
K	3.89 (3.64, 4.13)	3.87 (3.63, 4.11)	3.91 (3.67, 4.14)	0.012
Ca	2.28 (2.22, 2.34)	2.28 (2.23, 2.34)	2.29 (2.23, 2.35)	0.046
PT	10.90 (10.50, 11.50)	10.90 (10.40, 11.40)	10.90 (10.40, 11.50)	0.46
INR	0.95 (0.90, 1.00)	0.94 (0.90, 0.99)	0.95 (0.90, 1.00)	0.476
PTA	114.00 (101.00, 127.00)	114.60 (101.00, 128.00)	114.00 (101.00, 126.00)	0.9
TT	18.62 (17.80, 19.50)	18.62 (17.80, 19.50)	18.62 (17.80, 19.50)	0.894
APTT	25.90 (23.50, 28.70)	25.84 (23.50, 28.50)	26.10 (23.70, 28.80)	0.098
PF	3.09 (2.61, 3.88)	3.07 (2.60, 3.82)	3.17 (2.65, 3.93)	0.038
NLR	2.17 (1.65, 2.94)	2.10 (1.58, 2.96)	2.16 (1.64, 2.94)	0.342
PLR	141.39 (111.86, 182.49)	142.01 (109.91, 185.92)	140.43 (109.63, 180.43)	0.769
LMR	4.00 (3.02, 5.11)	4.02 (3.00, 5.18)	4.00 (3.01, 5.11)	0.781
ELR	0.11 (0.07, 0.17)	0.11 (0.06, 0.17)	0.11 (0.07, 0.18)	0.05
dNLR	1.52 (1.20, 1.98)	1.49 (1.16, 1.96)	1.53 (1.20, 1.99)	0.465
NLPR	0.01 (0.01, 0.01)	0.01 (0.01, 0.01)	0.01 (0.01, 0.01)	0.235
SII	577.34 (409.40, 831.31)	558.10 (403.46, 846.92)	588.33 (411.79, 846.68)	0.505
AISI	273.61 (173.56, 444.33)	265.11 (172.36, 456.99)	278.18 (176.70, 462.95)	0.67
SIRI	1.01 (0.70, 1.58)	0.98 (0.68, 1.61)	1.02 (0.70, 1.57)	0.518
LCR	0.75 (0.26, 1.72)	0.81 (0.24, 1.85)	0.74 (0.25, 1.63)	0.502
CRP/ALB	0.06 (0.03, 0.18)	0.06 (0.03, 0.18)	0.07 (0.03, 0.18)	0.4
p_CRP	2.44 (0.85, 7.78)	2.47 (0.85, 7.92)	2.56 (0.97, 8.20)	0.247
p_SAA	10.00 (7.00, 25.00)	11.00 (6.00, 24.00)	10.55 (7.00, 31.00)	0.451
p_PCT	0.05 (0.03, 0.09)	0.05 (0.03, 0.08)	0.05 (0.03, 0.09)	0.136
p_IL-6	14.03 (6.38, 36.84)	15.24 (6.66, 37.52)	15.60 (7.14, 37.08)	0.489
p_WBC	8.14 (6.41, 10.30)	8.13 (6.43, 10.34)	8.19 (6.46, 10.36)	0.898
p_Neut%	71.70 (62.40, 81.60)	71.20 (62.30, 82.00)	71.50 (62.00, 81.15)	0.99
p_Lymph%	19.70 (12.50, 27.55)	20.10 (12.30, 27.72)	19.50 (12.80, 27.80)	0.908
p_Mono%	5.60 (3.50, 7.10)	5.50 (3.50, 7.00)	5.60 (3.70, 7.10)	0.404
p_Eos%	1.40 (0.60, 2.70)	1.40 (0.58, 2.60)	1.50 (0.60, 2.80)	0.278

p_Baso%	0.40 (0.30, 0.60)	0.40 (0.30, 0.60)	0.40 (0.20, 0.60)	0.495
p_Neut	5.66 (4.16, 7.89)	5.66 (4.17, 7.91)	5.73 (4.18, 7.93)	0.911
p_Lymph	1.50 (1.02, 2.11)	1.53 (1.05, 2.10)	1.51 (1.03, 2.06)	0.952
p_Mono	0.43 (0.27, 0.60)	0.42 (0.26, 0.59)	0.43 (0.28, 0.59)	0.476
p_Eos	0.11 (0.05, 0.21)	0.11 (0.04, 0.20)	0.11 (0.05, 0.21)	0.247
p_Baso	0.03 (0.02, 0.04)	0.03 (0.02, 0.05)	0.03 (0.02, 0.04)	0.575
p_NRBC	0.00 (0.00, 0.00)	0.00 (0.00, 0.00)	0.00 (0.00, 0.00)	0.36
p_NRBC/RBC	0.00 (0.00, 0.00)	0.00 (0.00, 0.00)	0.00 (0.00, 0.00)	0.262
p_RBC	4.36 (3.89, 4.83)	4.37 (3.93, 4.82)	4.32 (3.86, 4.80)	0.153
p_HGB	123.00 (110.00, 137.00)	124.00 (110.00, 137.00)	123.00 (109.00, 136.00)	0.186
p_HCT	0.37 (0.34, 0.41)	0.38 (0.34, 0.41)	0.37 (0.34, 0.41)	0.219
p_MCV	87.70 (83.30, 91.20)	87.50 (83.10, 90.90)	87.30 (83.00, 91.00)	0.321
p_MCH	29.30 (27.40, 30.60)	29.20 (27.40, 30.50)	29.10 (27.40, 30.50)	0.388
p_MCHC	331.00 (320.00, 340.00)	331.00 (320.00, 341.00)	331.00 (320.00, 340.00)	0.899
p_RDW	12.70 (12.00, 13.70)	12.60 (12.00, 13.60)	12.70 (12.10, 13.70)	0.273
p_PLT	250.00 (208.00, 301.00)	250.00 (208.00, 303.00)	254.00 (212.00, 302.00)	0.363
p_MPV	9.70 (9.20, 10.20)	9.70 (9.20, 10.30)	9.70 (9.20, 10.30)	0.787
p_Plateletcrit	0.24 (0.20, 0.29)	0.24 (0.20, 0.29)	0.25 (0.21, 0.29)	0.222
p_BUA	264.10 (197.90, 338.95)	256.00 (195.00, 333.00)	265.50 (201.77, 336.88)	0.424
p_BUN	4.20 (3.30, 5.40)	4.09 (3.25, 5.22)	4.20 (3.30, 5.50)	0.165
p_Scr	94.00 (76.00, 118.00)	93.00 (75.00, 118.00)	94.00 (76.00, 120.25)	0.393
p_eGFR	75.44 (55.38, 94.77)	77.22 (58.43, 95.89)	74.32 (55.15, 93.58)	0.15
p_HCO3	23.10 (21.20, 24.90)	23.00 (21.10, 24.80)	22.90 (21.00, 24.80)	0.108
p_β2_MG	2.13 (1.68, 2.84)	2.05 (1.62, 2.77)	2.14 (1.69, 2.94)	0.009
p_TP	65.50 (61.50, 69.20)	65.40 (61.80, 69.50)	65.50 (61.20, 69.50)	0.713
p_ALB	36.30 (33.90, 38.70)	36.40 (33.90, 38.80)	36.20 (33.40, 38.60)	0.401
p_GLB	28.80 (26.10, 31.80)	28.90 (26.20, 32.10)	28.90 (26.10, 32.10)	0.518
p_ALB/GLB	1.30 (1.10, 1.40)	1.30 (1.10, 1.40)	1.30 (1.10, 1.40)	0.337
p_BIL	10.10 (7.30, 13.60)	10.20 (7.80, 14.10)	10.00 (7.30, 14.00)	0.265

p_DBIL	4.60 (3.50, 6.00)	4.70 (3.50, 6.10)	4.60 (3.50, 6.20)	0.534
p_IBil	5.40 (3.60, 7.60)	5.50 (3.80, 7.90)	5.40 (3.60, 7.90)	0.151
p_GPT	14.00 (10.00, 21.00)	14.00 (10.00, 21.00)	14.00 (10.00, 21.00)	0.594
p_GOT	16.25 (14.00, 20.20)	16.80 (14.00, 20.00)	16.00 (13.90, 20.83)	0.815
p_ALP	65.00 (54.00, 77.00)	63.00 (53.00, 76.00)	65.00 (54.00, 77.00)	0.189
p_GGT	25.00 (16.90, 40.40)	24.50 (17.00, 39.00)	24.40 (17.00, 39.00)	0.929
p_TBA	1.80 (1.00, 3.40)	1.70 (1.00, 3.30)	1.80 (0.90, 3.20)	0.318
p_K	3.84 (3.57, 4.11)	3.80 (3.58, 4.10)	3.85 (3.57, 4.12)	0.333
p_Ca	2.18 (2.10, 2.26)	2.18 (2.09, 2.26)	2.18 (2.09, 2.26)	0.446
p_PT	11.70 (11.20, 12.30)	11.70 (11.20, 12.20)	11.80 (11.30, 12.30)	0.206
p_INR	1.02 (0.97, 1.07)	1.02 (0.97, 1.06)	1.03 (0.98, 1.07)	0.199
p_PTA	103.00 (91.00, 114.00)	103.00 (93.00, 114.00)	100.00 (91.00, 112.00)	0.079
p_TT	18.50 (17.58, 19.50)	18.60 (17.67, 19.50)	18.50 (17.60, 19.40)	0.508
p_APTT	26.40 (23.50, 29.50)	26.30 (22.80, 29.02)	26.90 (24.30, 30.00)	0.037
p_PF	3.11 (2.53, 3.93)	3.12 (2.57, 3.80)	3.15 (2.53, 3.93)	0.894
p_NLR	3.64 (2.28, 6.52)	3.54 (2.24, 6.76)	3.67 (2.24, 6.31)	0.938
p_PLR	165.97 (116.67, 247.78)	161.59 (116.03, 248.89)	169.87 (119.41, 246.23)	0.759
p_LMR	3.97 (2.60, 6.03)	4.07 (2.64, 6.23)	3.93 (2.60, 5.93)	0.352
p_ELR	0.07 (0.04, 0.13)	0.07 (0.03, 0.12)	0.07 (0.04, 0.13)	0.103
p_dNLR	2.53 (1.66, 4.44)	2.46 (1.65, 4.55)	2.51 (1.63, 4.30)	0.991
p_NLPR	0.01 (0.01, 0.03)	0.01 (0.01, 0.03)	0.01 (0.01, 0.03)	0.942
p_SII	925.00 (551.56, 1674.08)	894.91 (540.06, 1786.22)	950.06 (560.46, 1664.66)	0.82
p_AISI	336.41 (188.84, 668.96)	328.32 (175.59, 615.35)	354.94 (193.64, 644.39)	0.3
p_LCR	0.59 (0.16, 1.87)	0.61 (0.16, 1.80)	0.59 (0.15, 1.63)	0.4
p_SIRI	1.32 (0.77, 2.47)	1.30 (0.74, 2.44)	1.35 (0.78, 2.50)	0.453
p_CRP/ALB	0.07 (0.02, 0.23)	0.07 (0.02, 0.22)	0.07 (0.03, 0.23)	0.532
p_PCT/ALB	<0.01 (<0.01, <0.01)	<0.01 (<0.01, <0.01)	<0.01 (<0.01, <0.01)	0.063
WBC-p_WBC	0.86 (0.68, 1.08)	0.86 (0.68, 1.06)	0.87 (0.69, 1.09)	0.412

U_LEU, urine leukocytes; U_NIT, urine nitrite; U_PRO, urine protein; SED_WBC, sediment white blood cells; SED_bacteria, sediment bacteria; WBC, white blood count; Neut%, neutrophil percentage; Neut, absolute neutrophil count; Mono, monocyte count; RDW, red

cell distribution width; β2 MG, β2 microglobulin; CysC, cystatin C; TP, total protein; GLB, globulin; ALP, alkaline phosphatase; GGT, gamma-glutamyl transferase; SAA, Serum amyloid A; PCT, Procalcitonin; IL-6, Interleukin-6; CRP, C-reactive protein; PT, prothrombin time; INR, international normalized ratio; PF, partial thromboplastin time; SED_EC, sediment erythrocytes; Lymph%, lymphocyte percentage; HGB, hemoglobin; HCT, hematocrit; MCH, mean corpuscular hemoglobin; eGFR, estimated glomerular filtration rate; ALB, albumin; IBil, indirect bilirubin; NLR, neutrophil-to-lymphocyte ratio; dNLR, derived NLR; PLR, platelet-to-lymphocyte ratio; LMR, lymphocyte-to-monocyte ratio; ELR, eosinophil-to-lymphocyte ratio; NLPR, neutrophil-lymphocyte-platelet ratio; SII, systemic immune-inflammation index; AISI, aggregate index of systemic inflammation; SIRI, systemic inflammation response index; LCR, lymphocyte-to-CRP ratio; CRP/ALB, CRP-to-albumin ratio; p_, postoperative.
